# The Tandem Reconnection and Cusp Electrodynamics Reconnaissance Satellites (TRACERS) Mission

**DOI:** 10.1007/s11214-025-01184-4

**Published:** 2025-06-27

**Authors:** D. M. Miles, C. A. Kletzing, S. A. Fuselier, K. A. Goodrich, J. W. Bonnell, S. Bounds, H. Cao, I. H. Cairns, L. J. Chen, I. W. Christopher, K. Cleveland, H. K. Connor, D. Crawford, J. Dolan, J. C. Dorelli, R. Dvorsky, M. G. Finley, R. H. W. Friedel, J. S. Halekas, G. B. Hospodarsky, A. N. Jaynes, J. Labelle, Y. Lin, M. Øieroset, S. M. Petrinec, M. L. Phillips, B. Powers, R. Prasad, A. Rospos, O. Santolik, R. J. Strangeway, K. J. Trattner, A. Washington

**Affiliations:** 1 Department of Physics and Astronomy, University of Iowa, Iowa City, IA USA; 2 Southwest Research Institute, San Antonio, TX USA; 3 University of Texas at San Antonio, San Antonio, TX USA; 4 Department of Physics and Astronomy, West Virginia University, Morgantown, WV USA; 5 Space Sciences Laboratory, University of California, Berkeley, CA USA; 6 Department of Physics, University of Sydney, Sydney, New South Whales Australia; 7 NASA Goddard Space Flight Center, Greenbelt, MD USA; 8 Cleveland Aerospace Technology Services, Davidsonville, MD USA; 9 Department of Astronomy, University of Maryland, College Park, MD USA; 10 NASA Headquarters, Washington, Department of Astronomy, University of Maryland, College Park, MD USA; 11 Department of Physics, Dartmouth College, Hanover, NH USA; 12 Department of Physics, Auburn University, Auburn, AL USA; 13 Lockheed Martin Advanced Technology Center, Palo Alto, CA USA; 14 Millenium Space Systems, A Boeing Company, El Segundo, CA USA; 15 Department of Space Physics, Institute of Atmospheric Physics of the Czech Academy of Sciences, Prague, Czechia; 16 Faculty of Mathematics and Physics, Charles University, Prague, Czechia; 17 Department of Earth, Planetary and Space Sciences, University of California Los Angeles, Los Angeles, CA USA; 18 Laboratory of Atmospheric and Space Sciences, University of Colorado Boulder, Colorado, Boulder, USA

**Keywords:** TRACERS, Cusp, Magnetic reconnection, NASA, Heliophysics

## Abstract

The overarching science goal of the Tandem Reconnection And Cusp Electrodynamics Reconnaissance Satellites (TRACERS) mission is to connect the cusp to the magnetosphere by discovering how spatial or temporal variations in magnetic reconnection drive cusp dynamics. This goal will be achieved with a simple mission design comprising two identical small spacecraft in identical low-Earth orbits in a follow-the-leader configuration. TRACERS will make repeated measurements in the cusp for a twelve-month primary mission using plasma and field instruments. These data will be analyzed using established dual-spacecraft techniques and supported by modeling that ensures science closure on the objectives. The TRACERS team leverages hardware collaborations from the University of Iowa, Southwest Research Institute, University of California Los Angeles, University of California Berkeley, and Millennium Space Systems. The larger science team consists of experts in reconnection, cusp physics, and modeling. TRACERS is dedicated to its proposer, and original Principal Investigator, Professor Craig Kletzing.

## Introduction

The overarching science goal of the Tandem Reconnection And Cusp Electrodynamics Reconnaissance Satellites (TRACERS) mission is to connect the cusp to the magnetosphere by discovering how spatial or temporal variations in magnetic reconnection drive cusp dynamics. This goal is achieved through three Science Objectives (SO1-3):

Science Objective 1. Determine whether magnetopause reconnection is primarily spatially or temporally variable for a range of solar wind conditions.

Science Objective 2. For temporally varying reconnection, determine how the reconnection rate evolves.

Science Objective 3. Determine to what extent dynamic structures in the cusp are associated with temporal versus spatial reconnection.

TRACERS aims to answer the fundamental question of magnetic reconnection posed more than two decades ago: Is reconnection dominantly spatially or temporally variable? This key question, when answered, determines the structure of one of the most basic features of the Earth’s magnetosphere. Now is the ideal time to answer this essential question because of the extensive work being done on the microphysics of reconnection that is motivated by the Magnetospheric Multiscale (MMS) mission. TRACERS completes the picture of reconnection, bridging large to small scales by investigating reconnection variability in the Earth’s northern magnetospheric cusp.

**Fig. 1:**
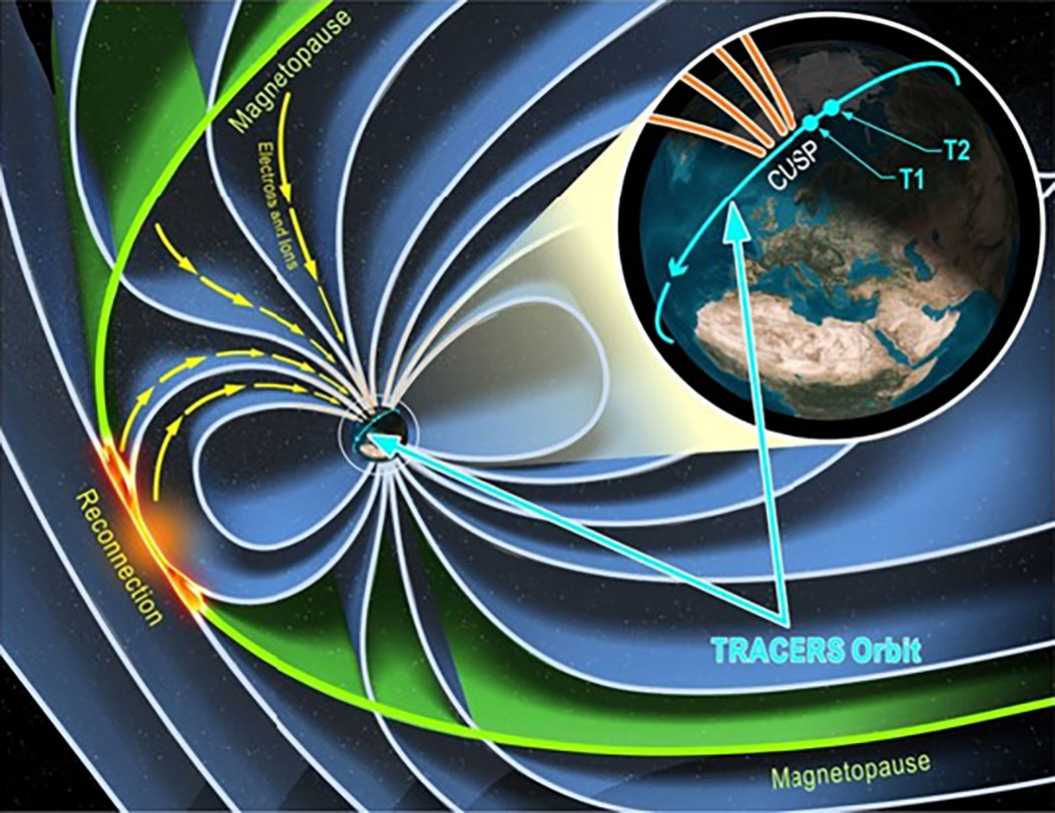
Magnetic reconnection modifies the magnetic field lines that thread the cusp. TRACERS takes advantage of multiple passes of two spacecraft through the cusp to observe these modifications and achieve its overarching science goal

The northern and southern cusps act as funnels, concentrating the magnetic field lines that thread the entire dayside magnetopause. Spacecraft that fly through the cusp at low altitudes observe this concentration of magnetic field lines and the effects that reconnection has on them (Fig. 1). Multiple spacecraft missions have flown through the cusp at many altitudes and demonstrated that reconnection is both spatially and temporally variable. What has eluded magnetospheric physics is whether one or the other of these types of variability is dominant, and if there are specific solar wind conditions that control this dominance. This determination will be accomplished with a dedicated mission that makes many passes through the cusp, spanning a wide range of solar wind conditions. Furthermore, this mission requires two spacecraft in the same orbit that are close together in time to discriminate between temporal and spatial changes. This is the essence of the TRACERS mission. TRACERS uses two innovations to accomplish its overarching science goal: 1) the two identical spinning TRACERS spacecraft are in the same Sun-synchronous orbit allowing them to separate spatial and temporal variations in reconnection and 2) the two spacecraft keep their spin axes oriented nearly along the magnetic field in the cusp (with minor slews once per day), significantly simplifying the instrumentation and enabling precise electric and magnetic field and particle measurements in the cusp that are needed to resolve reconnection variability.

TRACERS consists of two identical spacecraft, a first for a NASA Small Explorer (SMEX) mission, in a circular, Sun-synchronous orbit (SSO) that transits the cusp >3000 times during the 12-month primary science mission. The inter-spacecraft, along-track separation varies from 10 to 120 seconds (75 to 900 km, or 0.1 to 1 deg along track), through the course of the mission. The spacecraft have identical instrument suites consisting of the Analyzer for Cusp Ions (ACI), a ‘top-hat’ toroidal electrostatic analyzer provided by the Southwest Research Institute (Fuselier et al. 2025, this collection); the Analyzer for Cusp Electrons (ACE), a ‘top-hat’ electrostatic analyzer provided by the University of Iowa (Halekas et al. 2025, this collection); the Electric Field Instrument (EFI), a double probe experiment with Stacer booms provided by the University of California Berkeley (Bonnell et al. 2025, this collection); the Magnetic Search Coil (MSC), a low frequency search coil magnetometer provided by the University of Iowa (Hospodarsky et al. 2025, this collection); the Magnetometer (MAG), a DC fluxgate magnetometer provided by the University of California Los Angeles (Strangeway et al. 2025, this collection); and MAGnetometers for Innovation and Capability (MAGIC), a technology demonstration of a new fluxgate magnetometer technology provided by the University of Iowa (Miles et al. 2025, this collection).

TRACERS uses a single science operations mode to make the required observations in the science Region of Interest (ROI), defined as the part of the orbit from 85° to 60° North magnetic latitude that is expected to contain the cusp. The other primary operational state is the Back Orbit (BOR), with a greatly reduced data volume telemetered to enable in-situ instrument calibration, and in which the spacecraft makes orbit or attitude adjustments. These two very simple operational states, the ROI and the BOR, provide efficient use of resources and simplify mission operations. Data are downlinked daily to the Mission Operations Center (MOC) at Millenium Space Systems, mirrored by the Science Operations Center (SOC) at the University of Iowa, and distributed to the science team for analysis and then to the public for open access. These measurements are complemented by a robust theory and modeling effort, which allows placement of the observations in a global context and to understand the underlying physical processes of cusp electrodynamics.

### In Memoriam: Professor Craig Kletzing

**Fig. 2:**
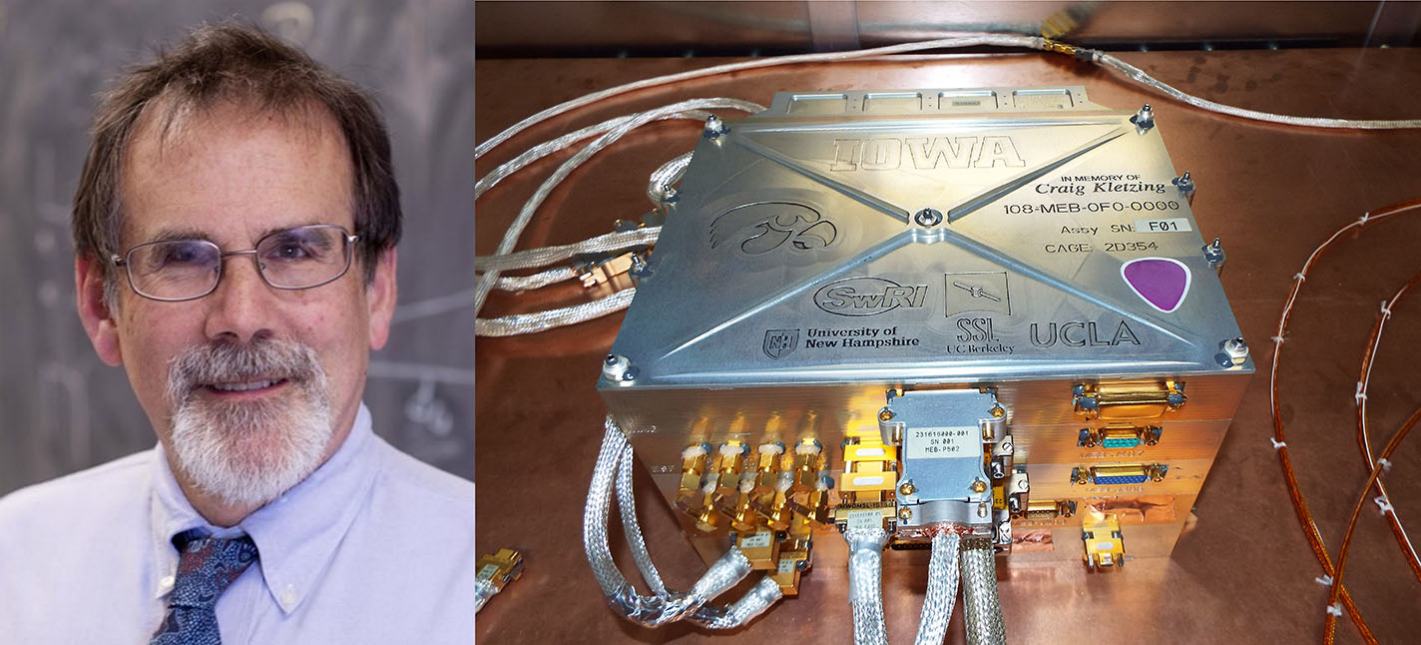
TRACERS is dedicated to Prof. Craig Kletzing who died peacefully at home on August 10, 2023. The instrument suite main electronics box on each spacecraft includes one of Craig’s guitar picks (purple). TRACERS will carry Craig’s curiosity into space one last time

TRACERS is dedicated to its proposer and original Principal Investigator, Professor Craig Kletzing (Fig. 2), who died peacefully at home on August 10, 2023. Craig had a decorated career in experimental space plasma physics and was an enthusiastic educator and mentor who left a legacy of students and collaborators. TRACERS will carry Craig’s curiosity into space one last time.

## Inspiration from the TRICE-2 Sounding Rocket Missions

The pathfinder for the TRACERS mission was the Twin Rockets to Investigate Cusp Electrodynamics 2 (TRICE-2) mission. The high-flyer and low-flyer pair of rockets was launched on December 8, 2018, into the northern magnetospheric cusp to study the spatial and temporal nature of cusp structures. TRICE-2 carried the progenitor instruments to the TRACERS instrument suite, providing a robust single case-study of the data that will be available statistically from the TRACERS mission. The TRICE-2 observations are presented by Trattner et al. (2025, this collection).

## Science Objectives

Magnetic reconnection is a fundamental process that occurs throughout the universe. It converts magnetic energy into particle energy and is the energy source of solar flares, substorms in the Earth’s magnetotail, and particle acceleration at the Earth’s magnetopause. It is also the primary process for the transport of plasma from the magnetosheath into the magnetosphere. Although a long-standing area of research in space physics and a Decadal Survey Critical Science Goal (Heliophysics Committee on a Decadal Strategy for Solar and Space Physics 2013), two key unanswered questions have remained:1) How is reconnection initiated and sustained? and 2) Is reconnection dominantly spatially or temporally variable? The second question is the focus of the TRACERS mission.

The first question, understanding how reconnection is initiated, is a basic plasma physics problem which requires detailed in situ study at very small spatial scales in a very constrained region at the Earth’s magnetopause and in the magnetotail, and is being answered by the multi-spacecraft Magnetospheric Multiscale (MMS) mission (Burch et al. 2016; Fuselier et al. 2016). The second question, determining whether reconnection is dominantly spatially or temporally variable, addresses where and how reconnection takes place on the global scale of the entire magnetosphere. Spacecraft like MMS cross the reconnection regions rapidly at a single position and revisit the regions at widely separated times, making it difficult to discern spatial or temporal reconnection structures on a regular basis. In contrast, TRACERS answers this second question using sustained measurements of spatial and temporal reconnection rate variability that cannot be obtained through in situ measurements at the magnetopause or in the magnetotail.

**Fig. 3:**
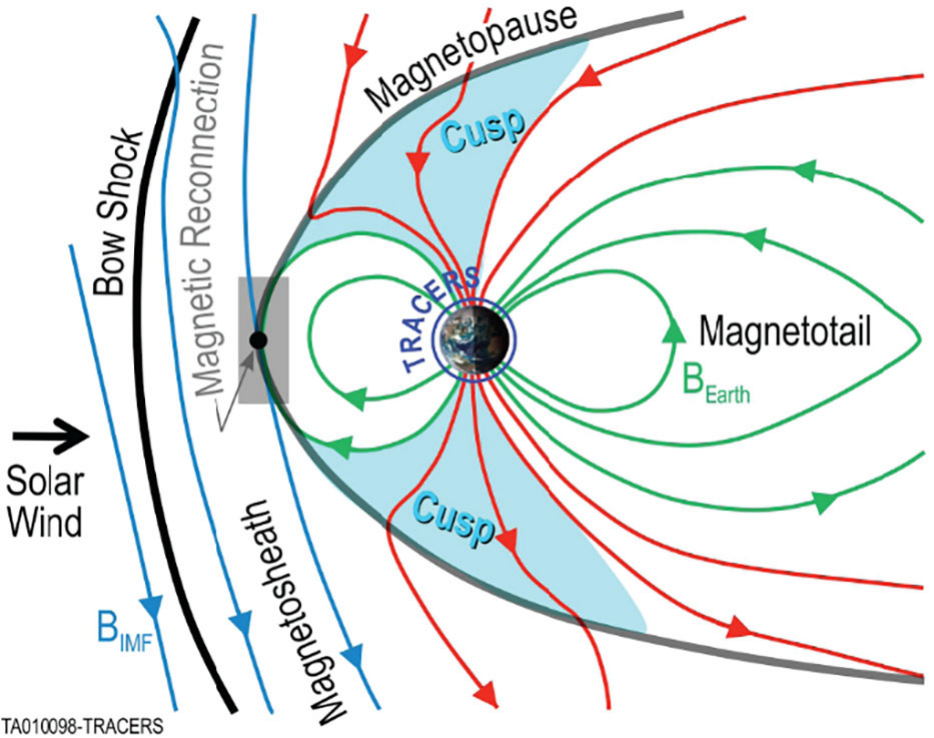
The Earth’s magnetospheric cusps concentrate magnetic reconnection effects into a small, low altitude region. TRACERS flies through this region, separating temporal and spatial effects

It has been known for more than 25 years that the only places in the magnetosphere where sustained measurements of spatial and temporal variability in reconnection can be obtained are in Earth’s magnetospheric cusps (shaded regions in Fig. 3). The cusps act like funnels that concentrate the temporal and spatial effects of reconnection into a very small region at low altitudes. With two spacecraft in low-Earth orbit, TRACERS samples the low-altitude northern cusp on a regular basis and separates temporal and spatial effects to answer this important, decades-old question (Onsager and Elphic 1996) of the nature of reconnection variability. While the southern cusp is equally viable for TRACERS science (Fig. 3), data volume and spacecraft attitude reorientation requirements limit observations to one cusp per orbit for the twelve-month primary science mission. The team chose the northern cusp because there are significant complementary ground assets available (see Sect. 7).

The separation of temporal and spatial effects of magnetic reconnection has profound implications for the global transfer of mass, momentum, and energy from the solar wind into the magnetosphere. Reconnection is the primary transport process across the magnetopause. The degree of variability of its rate and how this rate depends on the reconnection location are critical unknowns. These must be determined to make substantive progress on solar wind-magnetosphere coupling beyond the simple assumption that the magnetopause reconnection rate is steady and uniform across the magnetopause.

### Science Strategy and Closure

Science Objective 1 uses a database of TRACERS cusp crossings to determine if reconnection variability is dominantly spatial or temporal for a range of solar wind conditions. Science Objective 2 takes this determination a step farther by quantifying the extent of reconnection rate variability. Science Objective 3 uses the full data set to determine to what extent a variety of cusp dynamic structures are associated with temporal and/or spatial reconnection variability. Finally, 3-D modeling completes the ionospheric cusp-magnetosphere connection. Together, Science Objectives 1, 2 and 3 provide significant advancement in our understanding of how reconnection behaves on a global scale.

The TRACERS mission design is simple, using two identical, small spacecraft that are launched into a common low-Earth orbit in a follow-the-leader configuration by a shared launch. The first-ever use of two closely separated spacecraft in the low-altitude cusp will unambiguously resolve spatial and temporal reconnection variability. TRACERS has a straight-forward mission operations strategy that uniquely focuses on the magnetospheric cusp and that does not change during the twelve months of primary science operations. TRACERS is predicted to repeat the same measurements in the northern cusp more than 3000 times using instruments designed specifically for low-altitude cusp observations. Most of these instruments have heritage from other magnetospheric missions and from the TRICE-2 rockets that launched in late 2018 (Di Mare and Howes 2024; Fuselier et al. 2022; Petrinec et al. 2023; Sawyer et al. 2021; Spicher et al. 2022; Trattner et al. 2024). The data analysis from these instruments uses well-established dual-spacecraft techniques and modeling to achieve science closure.

### Need and Value of TRACERS

The TRACERS overarching science goal and its three Science Objectives are directly aligned with two Heliophysics Decadal Survey (Heliophysics Committee on a Decadal Strategy for Solar and Space Physics 2013) - “Key Science Goal 2. Determine the dynamics and coupling of Earth’s magnetosphere, ionosphere, and atmosphere and their response to solar and terrestrial inputs” and “Key Science Goal 4. Discover and characterize fundamental processes that occur both within the heliosphere and throughout the universe.” Since reconnection is the dominant plasma transfer process at the magnetopause, the total amount of plasma transferred depends critically on whether reconnection is dominantly spatially or temporally variable. Understanding the modulation of reconnection is an essential element in understanding this fundamental process.

TRACERS also aligns with top-level objectives and research focus areas in: “Our Dynamic Space Environment: Heliophysics Science and Technology Roadmap for 2014-2033” (Heliophysics Roadmap Team 2014). Specifically:

- Solve the Fundamental Mysteries of Heliophysics (F)
  o Understand magnetic reconnection (F1)
  o Understand the plasma processes that accelerate and transport particles (F2)
- Understand the Nature of Our Home Planet (H)
  o Understand the coupling of the Earth’s magnetosphere-ionosphere-atmosphere system, and its response to external and internal forcing (H3).

TRACERS provides key measurements that advance the science of these objectives, making it a uniquely compelling mission.

### Science Objective 1: Determine Whether Magnetopause Reconnection Is Primarily Spatially or Temporally Variable for a Range of Solar Wind Conditions

**Fig. 4:**
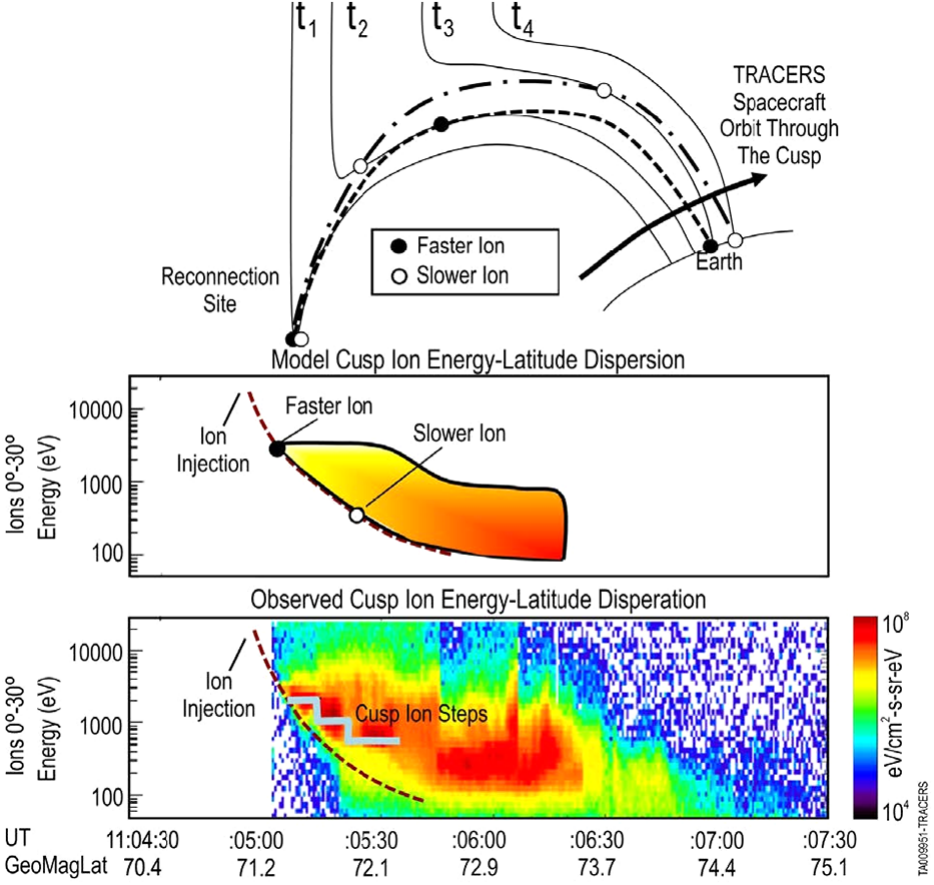
(top) Schematic of magnetopause reconnection for southward IMF. Dashed line shows faster ions while dashed-dot line shows slower ions. (middle) Modeled resulting cusp ion energy-latitude dispersion. (bottom) Equivalent observations from a low-altitude cusp crossing

Unlike reconnection in the Earth’s magnetotail, reconnection at the Earth’s magnetopause is believed to be driven by the solar wind dynamic pressure on the dayside (Fig. 4, top). This driven reconnection is believed to be continuously occurring somewhere on the magnetopause (see review by Cassak and Fuselier 2016). If there were no variability in reconnection (either temporally or spatially), then spacecraft flying through the cusp would observe a continuous, smoothly varying ion dispersion across a range of latitudes as shown in Fig. 4 (middle). However, observations such as Fig. 4 (bottom) show that abrupt changes in the ion dispersion, called cusp ion steps, are often observed (e.g., Lockwood et al. 1994; Lockwood and Smith 1992). These cusp ion steps indicate reconnection variability and, since reconnection is the primary solar wind-magnetosphere coupling process, understanding this variability is critical to understanding this coupling.

Figure 4 gives a schematic of magnetopause reconnection for southward IMF, showing the modeled resulting cusp ion dispersion, and actual observations from a low-altitude crossing of the cusp. At time t1, magnetospheric and magnetosheath magnetic fields reconnect, injecting ions (and electrons) into the magnetosphere. At time t2, the faster ions have moved further Earthward than the slower ions and the reconnected field line has convected northward. At time t3, the faster ions have arrived at the ionosphere, but the slower ions do not arrive until time t4 when the field line has convected further northward. A consequence of this velocity filter effect is that ions continuously injected near the reconnection site (Fig. 4, middle panel) with a range of energies are dispersed in latitude (and time) along a smooth curve. This curve is observable by a low-altitude spacecraft in the cusp (Rosenbauer et al. 1975). The energy-latitude dispersion is reversed for northward IMF and reconnection at high latitudes. Actual spacecraft observations in Fig. 4 (bottom) do not always show a smooth, continuous dispersion with latitude. Step-like features suggest multiple ion injections (Newell and Meng 1991). These step-like features are interpreted as either temporal or spatial reconnection rate variability in the cusp that arise because spacecraft do not follow individual reconnected flux tubes. TRACERS uses two spacecraft to separate temporal and spatial variability of reconnection in the cusp.

These cusp ion steps indicate reconnection variability. However, the type of magnetic reconnection variability has been hotly debated. The steps have been interpreted in one of two ways, either as temporal variability in the reconnection rate (Escoubet et al. 1992; Lockwood et al. 1994; Lockwood and Smith 1989) or as quasi-steady reconnection from spatially variable reconnection locations at the magnetopause (Lockwood and Smith 1992; Onsager et al. 1995). The basis of these distinctly different interpretations of cusp observations is that spacecraft flying through the cusp sample field lines that have experienced or that map to regions with different temporal/spatial histories of reconnection. Thus, for a range of solar wind conditions, whether the reconnection process is temporally sporadic from a fixed spatial location, or temporally steady from a variety of spatial locations, or some combination of both remains unresolved and is the focus of Science Objective 1.

**Fig. 5:**
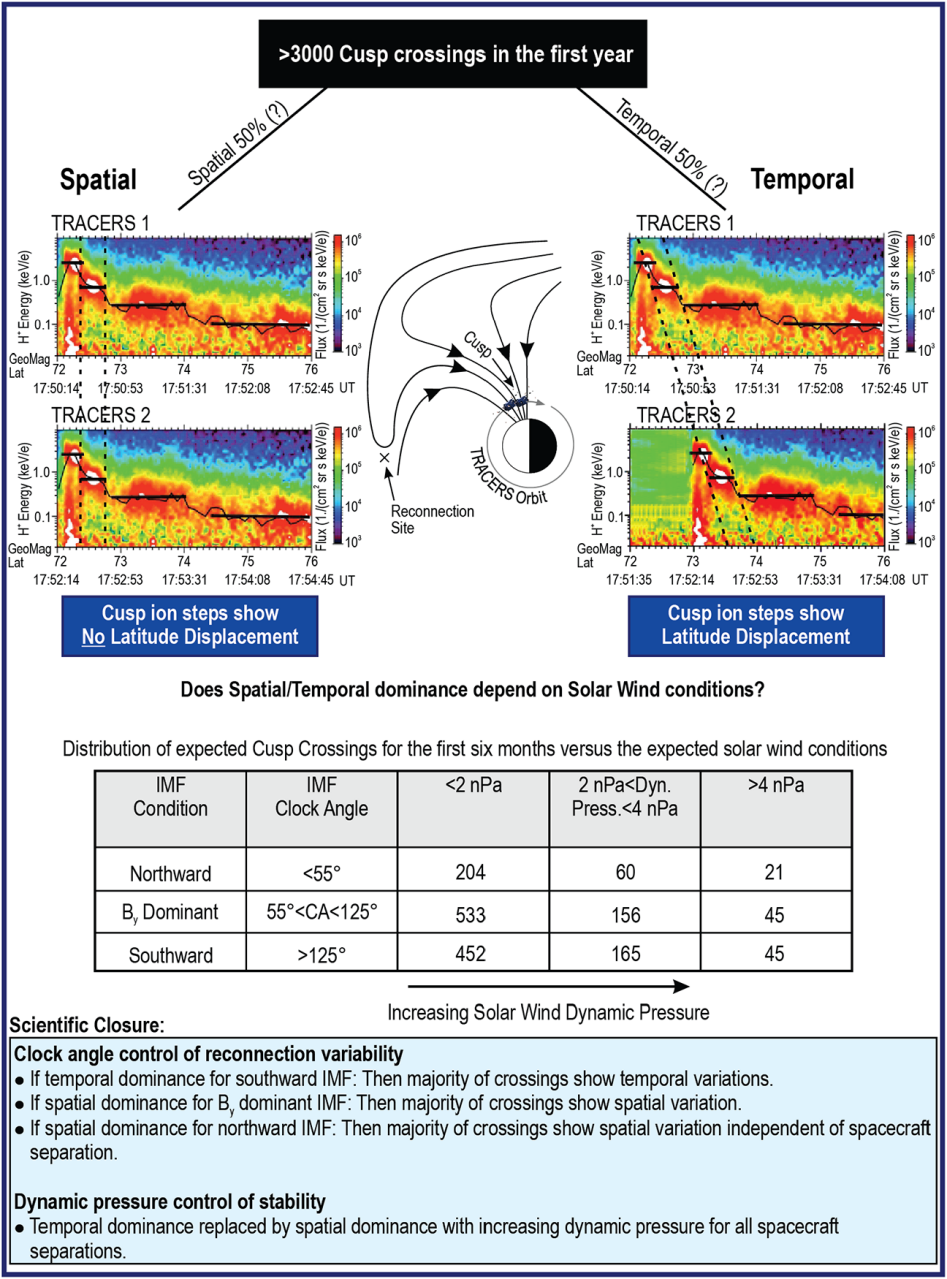
Science Objective 1 - Determine whether magnetopause reconnection is primarily spatially or temporally variable for a range of solar wind conditions

The key observational difference between the two types of variability is shown in Fig. 5. If temporal variation causes the steps, then ion dispersion features would be expected to continue convecting poleward and thus the steps move poleward as time progresses. In contrast, if spatial variation of steady reconnection causes the steps, then they do not convect but stay fixed in latitude because they are due to mapping of different locations to the spacecraft. By flying two identically instrumented spacecraft with close separation, TRACERS directly differentiates between these two cases.

Pilot studies of the dual-spacecraft technique proposed here have been tried using chance conjunctions between two spacecraft (Escoubet et al. 2006, 2008, 2013; Onsager et al. 1995; Trattner et al. 2002b,a, 2003, 2005; Wing et al. 2001). These chance conjunctions (about 9 total) demonstrated that some cusp ion steps are the result of spatial variation in reconnection (Onsager et al. 1995; Trattner et al. 2002a). Other observations, including combined Cluster spacecraft and SuperDARN radar observations, argue for temporal variation in reconnection (Boudouridis et al. 2001; Escoubet et al. 1992, 2006; Trattner et al. 2005, 2008). The difficulty with these previous multi-spacecraft studies has been that conjunctions in the cusp are rare. Furthermore, the spacecraft are often separated by large differences in space or time or are at relatively high altitude such that the spatial or temporal distinctions are not conclusive (Bosqued et al. 2005; Trattner et al. 2002a). These previous studies and recent global modeling of reconnection variability in the cusp (Connor et al. 2012, 2015; Tan et al. 2012) serve as a proof of concept to be exploited by the two-spacecraft TRACERS mission. They demonstrate the required measurements, inform the orbit’s desired altitude range and close spacecraft separations, and prove the necessary analysis methods for a dedicated cusp TRACERS mission.

The TRACERS orbit and a model of the cusp location (Petrinec et al. 2025, this collection) were used to predict ∼1650 crossings within the first 6 months of the mission. Thus, TRACERS produces a statistical data set more than 160 times larger and of higher quality than current published multiple spacecraft cusp crossings in the first 6 months. This large data set leaves no ambiguity in the type of reconnection variability occurring during a given cusp crossing for a given set of solar wind conditions. Figure 5 shows that, when observed by the two TRACERS spacecraft in succession, cusp ion steps that result from spatial variation in reconnection exhibit no latitude displacement. In contrast, cusp ion steps caused by temporal variation in reconnection at a single location exhibit latitude displacement from one spacecraft to the next. True latitude displacement is distinguished from a shift in the cusp by identifying the latitude of the open-closed field line boundary that marks the edge of the cusp. Figure 5 shows actual spacecraft data (from the FAST spacecraft) and these data have been offset by typical convection velocities in the cusp to illustrate this displacement.

The orbit altitude is important for unambiguous distinction of spatial and temporal cusp structures. Although there were several multi-spacecraft encounters of the cusp by the Cluster mission, these occurred at altitudes >6000 km where field line convection velocities are much larger than spacecraft velocities and spatial structures show temporal changes associated with large-scale motion of the cusp (Bosqued et al. 2005; Trattner et al. 2003). In contrast, the low altitude (∼590 km) TRACERS spacecraft cross the full latitudinal extent of the cusp in 30 s to 2 mins in a 96 min orbit. The along track spacecraft velocities (∼7.5 km/s) are much higher than the convection velocity of the cusp open-closed field line boundary (<1 km/s) or the convection velocity of open field lines (∼1 km/s). Thus, the TRACERS spacecraft always overtake cusp ion structures regardless of the direction of motion from north to south or south to north, making the distinction between spatial and temporal cusp structures unambiguous (Escoubet et al. 2013; Lockwood et al. 1994, 2001; Trattner et al. 2008).

The optimal minimum and maximum separation of the two TRACERS spacecraft along their common orbit as well as the required sampling rates for plasma convection and particle precipitation features depends on several factors and constraints: First, the motion of the open-closed field line boundary in response to changing solar wind conditions; second, the apparent duration of cusp ion steps in the spacecraft frame; third, the need to capture the transitions between successive cusp ion steps between the two spacecraft; and fourth, to resolve the precipitating electron signature and boundary convection velocity at the open-closed field line boundary. The change in latitude of the cusp for a 10 to 120 s along-track spacecraft separation and nominal field line convection of ∼1 km/s at ∼590 km altitude is 0.1 to 1 degree in latitude, with the 1-degree case depicted in Fig. 5. Cusp ion steps such as those shown in Fig. 5 have been observed to have durations of approximately 10-30 s or approximately 0.6 to 1.8 degrees in latitude. Therefore, the maximum spacecraft separation along track should not be greater than about 2 min so that specific cusp features are correlated between the spacecraft. In contrast, previous chance encounters with two spacecraft in the cusp were separated by tens of minutes or longer, leading to considerable ambiguity in the interpretation of the observations. The minimum separation should be greater than 10 s so that the trailing spacecraft encounters a step as the leading spacecraft is transitioning to the next ion step. To accurately characterize a 10 s step, the ion instrument should make about 20 measurements within the step, and thus should have time resolution of 0.5 s. Since the pitch angles of precipitating ions are important for determining the characteristics of cusp ion steps (Lockwood and Smith 1994), the ion instrument should measure 0-90° pitch angles and the magnetometer should have a sample rate commensurate with the ion energy sample rate.

The open-closed field line boundary is identified using the distinct electron signature of the loss of plasma sheet electrons and the appearance of energized magnetosheath electrons (e.g., Lockwood et al. 2005). A minimum time resolution of 0.5 s for each electron energy sweep is needed to determine this location for the minimum along-track separation of 10 s. The TRACERS 1 and 2 electric field measurements determine the motion of the cusp and the angle at which the spacecraft cross the region (Fig. 7). Similar (0.5 s) time resolution is required for these measurements.

### Science Objective 1 (SO1) Science Closure

The degree of temporal and spatial variability likely depends on solar wind conditions. Therefore, science closure is achieved by sorting cusp crossings by Interplanetary Magnetic Field (IMF) orientation (in four bins: southward, horizontal with $B_{y}$ dominant, northward, and ambiguous / dynamic) and dynamic pressure (in three bins of increasing pressure from nominal <2 nPa to high >4 nPa). IMF orientation and dynamic pressure are the two solar wind conditions that are known to affect the cusp the most (e.g., Fuselier et al. 2002; Lockwood and Smith 1994). This technique for predicting the statistics of conditions during a cusp crossing is analogous to the technique used successfully to predict the number of magnetopause encounters for the MMS mission (Fuselier et al. 2016; Petrinec et al. 2016). Solar wind conditions during the TRACERS mission are determined from a variety of solar wind monitors continuously maintained by NASA and NOAA.

In the first 6 months of the mission, TRACERS will have a statistically significant number of cusp crossings (200-300) for each interplanetary magnetic field (IMF) clock angle category for nominal (<2 nPa) solar wind dynamic pressure. This robust data set will be used to test the clock angle control of reconnection variability.

Temporal variation of reconnection is argued to occur most often for southward IMF conditions (e.g., Lockwood and Smith 1994). If this is true, then the majority of cusp crossings with southward IMF should exhibit temporal features. Spatial variation of reconnection is argued to occur when the observing spacecraft crosses reconnected flux tubes in the cusp obliquely (Trattner et al. 2003). If this is true, then the majority of cusp crossings when $B_{y}$ is dominant (and the cusp is oblique to the TRACERS orbit) should exhibit spatial features. Finally, continuous reconnection examples (where the reconnection rate appears to be quasi-steady, at least at 2-min time resolution) have been observed for northward IMF conditions (e.g., Frey et al. 2003). If this is true, then the majority of cusp crossings with northward IMF should exhibit spatial features.

Sorting the full twelve-month data set by dynamic pressure focuses on whether reconnection becomes unstable or stable when driving conditions at the magnetopause become strong. Magnetopause observations suggest that the reconnection rate saturates for southward IMF when the dynamic pressure is high (e.g., Anderson et al. 1997). If this is true, then cusp crossings with high dynamic pressure may be dominantly spatially variable whereas crossings with low dynamic pressure may be dominantly temporally variable. The same dependence on dynamic pressure may be true for northward IMF because continuous reconnection events for these conditions were observed when the dynamic pressure was high (Frey et al. 2003).

**Fig. 6:**
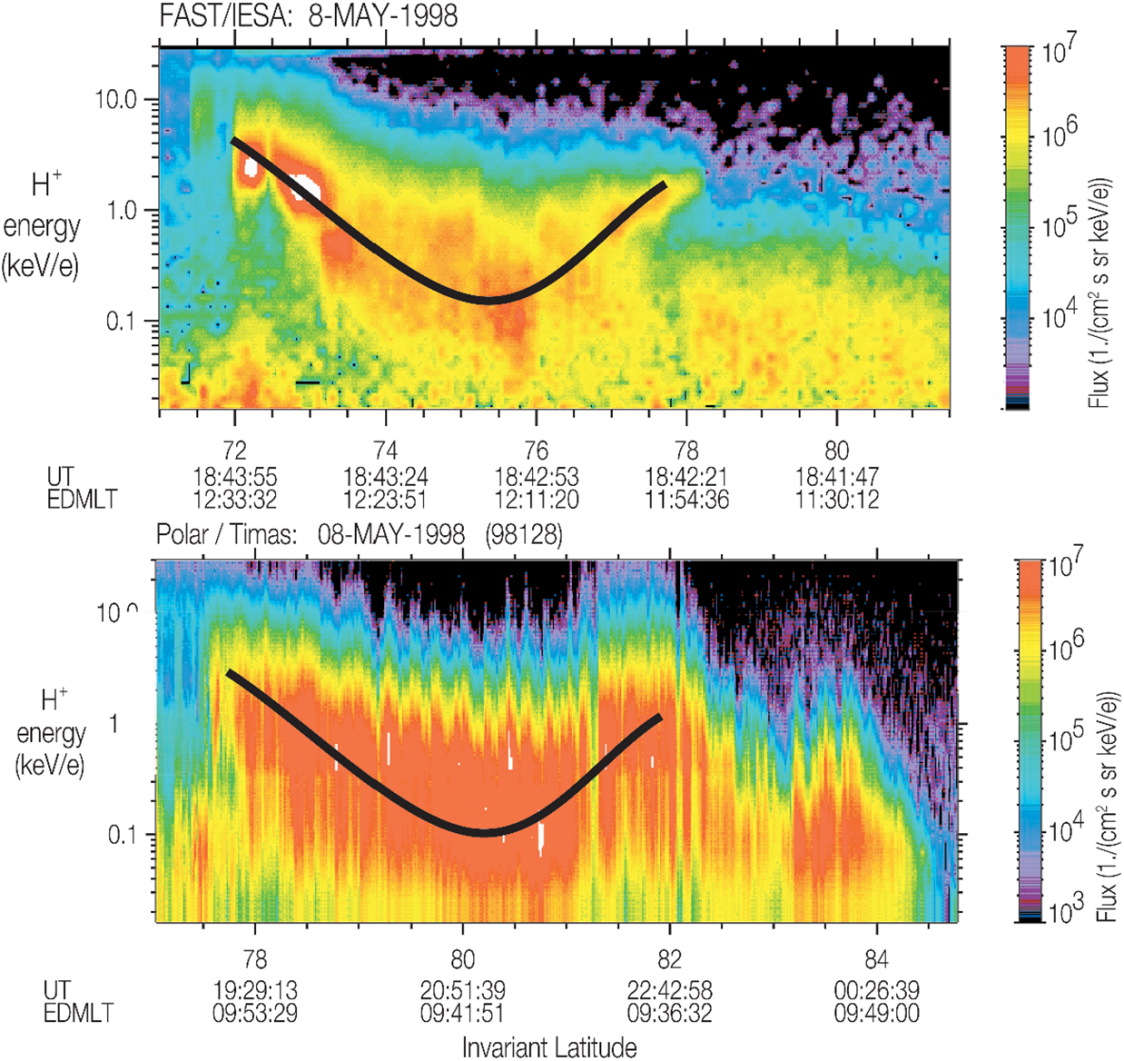
Fortuitous cusp conjunction between the Fast Auroral SnapshoT (FAST) and Polar spacecraft. The black overlaid line represents the average location of the maximum flux in the cusp ion energy dispersion. Although the spacecraft encountered the cusp at very different times and latitudes, the structure of the cusp dispersions is similar. While this suggests a spatial interpretation of the cusp ion dispersions, the results are ambiguous due to the large time separation between the spacecraft. Within individual cusp ion steps, there are more rapid ion dispersions suggesting that there is temporal variability within cusp ion steps. With superior time resolution and close spacecraft separations at low altitude, TRACERS will determine if ion dispersions within cusp ion steps are truly temporal variations in reconnection at timescales of seconds. (From Trattner et al. 2002b)

Finally, Trattner et al. (2002a) argue that even the spatially variable events may contain temporal variability within cusp ion steps and that reconnection may be temporally variable over a wide range of timescales (Fig. 6). The entire TRACERS data set will be analyzed for variability within the cusp ion steps, with particular focus on cusp crossings that are associated with spatially variable reconnection. With the 0.5 second time resolution requirement for the ions, the shortest duration ion steps contain 20 ion measurements and major changes in the ion energy distribution within the step will indicate temporal variability. Identifying these events will provide a measure of reconnection variability on timescales of seconds instead of variability on timescales of tens of seconds to minutes as in past studies of multi-spacecraft chance encounters of the cusp.

### Science Objective 2: For Temporally Varying Reconnection, Determine How the Reconnection Rate Evolves

Science Objective 2 quantifies the reconnection variability for the temporally varying events identified in Science Objective 1. The starting point for this objective is the set of events where temporal variability of reconnection has been established through Science Objective 1.

Newell and Meng (1995) performed a limited statistical analysis of cusp ion steps observed on single spacecraft and concluded that there could be large temporal variability in the reconnection rate, but that fully pulsed reconnection (i.e., where the reconnection electric field decreased to zero) is rare. Lockwood et al. (1994) developed a method for estimating the reconnection rate using data from a spacecraft cusp crossing. They argued that the reconnection rate varies dramatically by factors of 2 or more even for apparently steady-state, nearly step-free cusp dispersions. Thus, these two studies, which use essentially the same single-spacecraft data, come to dramatically different conclusions about time variability of the reconnection rate because cusp ion steps may be caused by spatial rather than temporal variations in reconnection (Onsager et al. 1995). The only way to resolve this controversy is to identify temporally variable reconnection events in the TRACERS two-spacecraft data set and then quantify the reconnection rate variability. Because mass and energy transfer via reconnection drives magnetospheric dynamics, the variability in the transfer is a critical unknown. For example, in the simple case that the reconnection rate is high and steady over a 2 min period across the dayside magnetopause, then the amount of plasma and energy transferred is 12 times greater than if the rate is high for 10 s and then at zero for the remaining 110 s. TRACERS, for the first time, will quantify the temporal variations of reconnection and therefore makes a quantitative determination of this crucial input that drives magnetospheric dynamics. Thus, TRACERS provides fundamental quantitative information on magnetospheric coupling, one of the most compelling aspects of magnetospheric interactions with the solar wind.

**Fig. 7:**
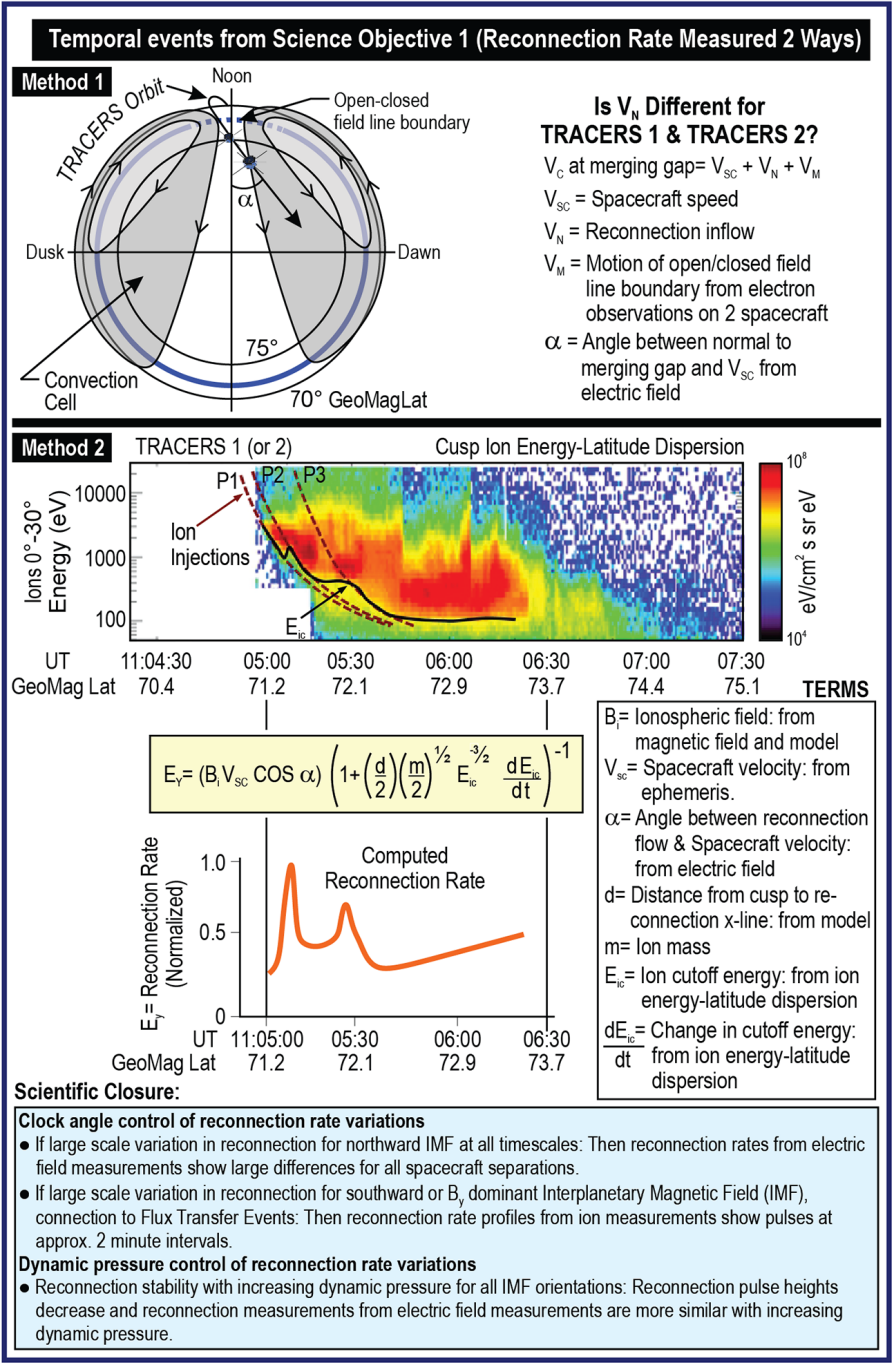
Science Objective 2 - For temporally varying reconnection, determine how the reconnection rate evolves

To answer Science Objective 2, reconnection rate variability will be computed using two independent methods. The first method is straightforward and yields two instantaneous measures of the reconnection electric field separated in time by the spacecraft separation. As the spacecraft cross the equatorward boundary of the cusp for southward IMF (Fig. 7), the electric field experiment measures the total convection velocity of the plasma across the open-closed field-line boundary. The velocity consists of the vector sum of the convection motion of the open-closed field line boundary and the reconnection inflow velocity. The convection motion of the open-closed field line boundary along the orbit is determined by comparing the boundary location as each spacecraft encounters it. The angle the orbit makes across the cusp is determined from electric field measurements. Subtracting off this convection motion, the remaining velocity vector is the sum of the motion of the open-closed field line boundary perpendicular to the orbit and the inflow velocity across the open-closed field line boundary. Assuming that the motion of the field line boundary perpendicular to the orbit is constant (a good assumption for the closely spaced TRACERS spacecraft), what remains are two instantaneous measurements of the ion inflow velocity across the open-closed field line boundary, which is the instantaneous reconnection rate (e.g., Lockwood et al. 2005). For every cusp crossing associated with temporal variability, TRACERS provides two instantaneous reconnection rate measurements separated by a time ranging from 10 s to 2 min. The rate measurements are not absolute numbers, but independent modeling of the cusp by the TRACERS modelling team reinforces the assumptions used to determine the reconnection rate and Science Objective 2 is achieved using only the relative changes in the rate from one spacecraft to the next.

The second method was pioneered by Lockwood et al. (1994) and applied to single spacecraft cusp observations. As shown in Fig. 7, Method 2 uses the low energy cutoff of the cusp ions to determine the time history of the reconnection rate as the spacecraft traverses the cusp. Plateaus and upshifts in the low energy cutoff indicate large changes in the reconnection rate, provided that the two-spacecraft measurements demonstrate that these changes are related to temporal and not spatial variability in reconnection. The first TRACERS spacecraft provides the time history of the reconnection rate during its cusp crossing. Modeling then provides the location of the reconnection site that is used to determine the magnitude of the rate. The second spacecraft follows the first up to two minutes later, providing a second time history of the reconnection rate. A key driver of this rate measurement is the energy resolution of the ion instrument. Observations shown in Fig. 7 for Science Objective 2 are from the Defense Meteorological Satellite Program (DMSP) spacecraft ion sensor and were taken at a similar altitude to that of TRACERS. These data demonstrate that logarithmically spaced energy steps with $\Delta $E/E not exceeding 22% (e.g., Knight et al. 2008) are sufficient to resolve the low energy cutoff of cusp ion precipitation.

**Fig. 8:**
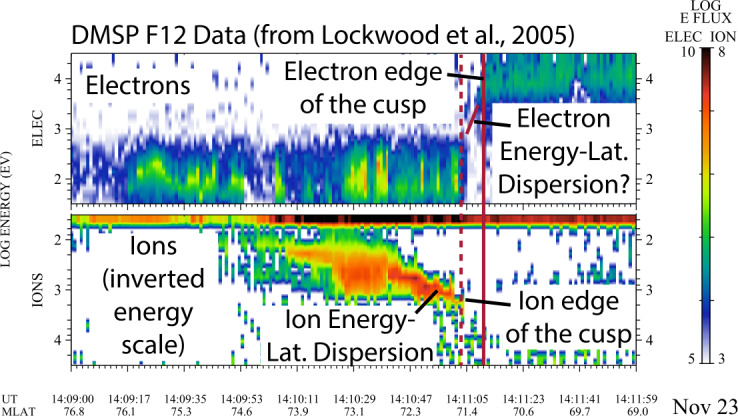
The same spatial and/or temporal reconnection variability and velocity dispersion effects that create structure in the ion precipitation should theoretically produce similar structure in the electron precipitation. Previous missions such as DMSP F12 (Lockwood et al. 2005) did not resolve structure in the electron edge; however, TRACERS’ enhanced time resolution and two spacecraft will enable discovery science targeting the electron edge of the Cusp

Figure 8 demonstrates the required energy range of the electron and ion instruments. The electron instrument must measure at least the lower edge of the magnetospheric electrons, which occur at ∼10 keV in Fig. 8 after 14:11:05 UT to distinguish the open-closed field line boundary. The instrument should measure the cusp electrons, which occur at 50-100 eV, at the low energy end. The same energy range is needed for the ions as for the electrons to distinguish the lower energy magnetospheric ions.

A unique feature of reconnection at the Earth’s magnetopause is the displacement of entering ions and electrons into the magnetosphere (Gosling et al. 1990; Øieroset et al. 2015). Electrons move much faster than ions, forming an inner edge to the low latitude boundary layer where heated magnetosheath electrons are observed but no magnetosheath ions are observed. For southward IMF, this electron edge produces a region that is inside the cusp, extending poleward from the open-closed field line boundary, but equatorward of the precipitating ions. It is bounded by the loss of ring current electrons at approximately 10 keV as they escape along open field lines and the appearance of the highest energy precipitating ions. The electron-only region lasts a few seconds and DMSP F12 does not resolve any potential electron structure within it. The same velocity dispersion effects and spatial or temporal reconnection variability that are responsible for structure in the ion precipitation should, in principle, produce similar structure in the electron precipitation in the electron edge. However, the spatial or temporal reconnection variability must be on timescales of less than a second since DMSP does not observe any electron dispersions in this case. TRACERS will search for precipitation structures in the electron edge of the cusp using its superior time resolution (<0.2 s) for electron measurements and two spacecraft to distinguish spatial from temporal variability. Identifying electron steps, plateaus, or rises in the lower edge of the electron dispersion would be significant discovery science and would be strong evidence for reconnection variability on timescales of less than a second.

### Science Objective 2 (SO2) Science Closure

The two methods for determining the reconnection rate described above do not yield the absolute reconnection rate. However, only the relative rate is needed to complete Science Objective 2. The scientific controversy focuses on very large (factors of 2 or more) changes in the reconnection rate (like those in Fig. 7). Modeling will assist in determining the absolute rate and take the results of Science Objective 2 beyond the study of reconnection as a fundamental process by determining how the point measurements of the rate relate to the global entry and acceleration of plasma into the magnetosphere.

The electric field experiment provides two time histories of the reconnection rate per cusp crossing with separation times between 10 s and 2 min. TRACERS will determine if these two time histories differ substantially depending on the time interval between measurements and on the solar wind conditions. When the IMF is northward, past cusp ion precipitation observations that consisted of 5-s snapshots at 2-min intervals argued for stable reconnection (Frey et al. 2003). The TRACERS time histories at a range of separation times may confirm this stability for a wide range of timescales or may find that the rate becomes variable at some shorter timescale. Either of these possible outcomes will provide new insight into reconnection at the high latitude magnetopause during northward IMF.

The continuous record of the reconnection rate that is provided by the ion measurements will conclusively determine the extent to which reconnection is temporally variable on timescales from seconds to minutes. This continuous record (Fig. 7) can demonstrate that fully or nearly-fully pulsed reconnection occurs at the magnetopause as argued by Lockwood et al. (1994). It has been argued further that the “pulse rate” observed in the cusp is commensurate with the few-minute recurrence rate for Flux Transfer Events at the magnetopause (Lockwood and Wild 1993). The two TRACERS spacecraft will conclusively determine if the two rates are similar for a wide range of IMF conditions.

Determining the amplitude of the pulses as a function of solar wind dynamic pressure will answer whether reconnection variability is reduced when the solar wind driver becomes stronger. There are no predictions for the change in occurrence rate of flux transfer events (i.e., reconnection rate variability) with dynamic pressure. Therefore, TRACERS will determine the connection by measuring changes in the pulse rate or amplitude changes with dynamic pressure over a wide range of IMF clock angles.

From Science Objective 1, there may be temporal variations within cusp ion steps. This variability indicates changes in the reconnection rate on timescales of seconds. The procedure for determining the reconnection rate for the entire cusp crossing is also directly applicable to plateaus or upshifts in the ion measurements within a cusp step. Thus, TRACERS will determine if spatially variable reconnection events contain considerable temporal changes in reconnection rate on timescales of seconds. Comparison with the results from the snapshots of reconnection rate from the electric field experiment within and across events will confirm the interpretation and provide a measure of the occurrence rate of such short timescale reconnection rate changes.

Finally, there may be events with electron steps in the electron edge of the cusp that are related to temporal reconnection rate variability on timescales less than a second. The procedure for determining the reconnection rate from the ion measurements is directly applicable to electron measurements. Thus, TRACERS may determine if there are large changes in the reconnection rate for a wide range of solar wind conditions on timescales from a fraction of a second to several minutes. Such a broad range of timescales covers the meaningful timescales from the convection time of electrons into and across the reconnection diffusion region (at fractions of a second) to the flux transfer event recurrence rate of about 2 minutes.

The detailed investigation of the reconnection rate that TRACERS provides in completing Science Objective 2 has important implications for magnetospheric dynamics. These implications are explored in Sect. 3.9 using global modeling of the reconnection process and the resulting cusp ion precipitation.

### Science Objective 3: Determine to What Extent Dynamic Structures in the Cusp Are Associated with Temporal Versus Spatial Reconnection

There are several dynamic structures or features observed in the cusp associated with temporal versus spatial reconnection, including Alfvén and other waves, electron precipitation, and ion outflows that complete the connection of the cusp to the magnetosphere. The first two of these are natural consequences of the reconnection processes and TRACERS will identify how these oft-observed features relate to the type of reconnection. Based on previous observations and theory, spatial reconnection events should have a distinct set of dynamic features associated with them while temporal events should have a different set and sequence of features. Understanding the relationship between dynamical features and spatial/temporal reconnection processes is important because these individual dynamical features are detectable by a wide range of ground-based or in situ techniques independent of the distinction between spatial and temporal reconnection effects in the cusp. TRACERS thereby motivates improved methods that can inexpensively and comprehensively monitor the nature of reconnection and plasma transfer into the magnetosphere.

At frequencies lower than the ion gyrofrequency, changes in the magnetic field topology of a plasma launch Alfvén waves. Magnetic reconnection can launch Alfvén waves, but these waves are also generated by variability in the solar wind impinging on the magnetopause, by the Kelvin-Helmholtz instability on the solar wind-magnetosphere interface, or by other instabilities intrinsic to the magnetosphere. Once generated, Alfvén waves propagate inward along geomagnetic field lines to the ionosphere. Along the way, phase mixing or instabilities can drive a cascade to shorter scales, and reflection effects can also structure the waves on narrow perpendicular scales (Chaston et al. 2006). Alfvén waves can drive wave-particle interactions on kinetic scales, resulting in dissipation of the wave energy (e.g., Chaston et al. 2000, 2007; Kletzing 1994; Kletzing and Hu 2001; and references therein). This dissipation produces time-dispersed electron bursts (Chaston et al. 2000; Chen et al. 2005; Kletzing and Hu 2001).

**Fig. 9:**
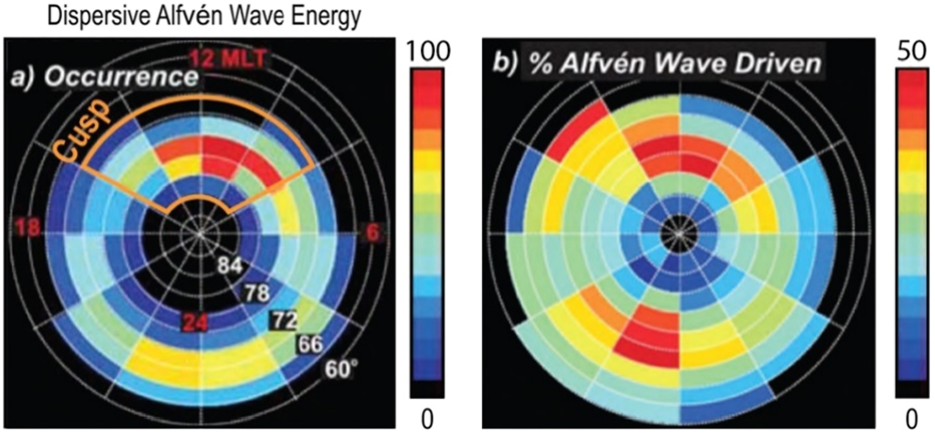
In-situ FAST measurements adapted from Chaston et al. (2007). Panel (a) occurrence frequency of Alfvén waves and panel (b) fraction of energetic electrons driven by the waves. If this statistical picture is consistent with individual cusp crossings, TRACERS should observe Alfvén waves nearly 100% of the time in the cusp. Where they occur relative to cusp ion steps determines if they are associated with temporal or spatial reconnection

Alfvén waves play an important role in cusp physics. Several spacecraft missions including FAST, Polar, and Akebono, as well as sounding rocket measurements, have observed these waves (e.g., Chaston et al. 1999; Keiling et al. 2001; Miyake et al. 2003; Tanaka et al. 2005a,b). Keiling et al. (2003) estimated that the Alfvén wave Poynting flux represents a significant fraction (∼30-35%) of the energy flux in the global ionospheric auroral luminosity. Chaston et al. (2007) used FAST data to directly compare statistical particle energy fluxes with statistical Poynting flux, binned according to Magnetic Local Time (MLT) and invariant latitude. Their key results with regards to TRACERS science are shown in Fig. 9. Panel (a) shows that Alfvén waves are nearly always present in the cusp with an occurrence frequency approaching 100%. Panel (b) shows that ∼50% of accelerated electrons are driven by the waves, or equivalently about half of Alfvén wave energy is converted to precipitating electron fluxes by the time the waves reach 4000 km altitude. These waves are also important because they participate in accelerating ions out of the ionosphere. These studies show that Alfvén wave signatures and their effects on particles are almost always present in the cusp and will be seen by TRACERS.

**Fig. 10:**
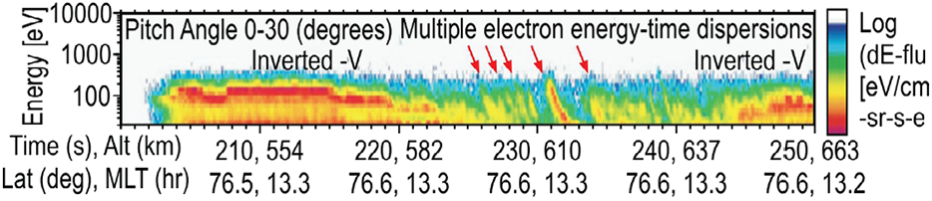
Energy-time spectrogram of precipitation cusp electrons adapted from Tanaka et al. (2005a). Time-dispersed electrons often associated with Alfvén waves are indicated by the red arrows. TRACERS ACE has the time resolution necessary to measure these time-dispersed electrons

Separate observations of Alfvén waves and particle signatures such as electron dispersion suggest a strong link between wave energy conversion and particle energization in the cusp. However, this link has not been established observationally. Part of the problem is that previous missions either had too low time resolution (e.g., DMSP) or flew at too high an altitude (e.g., FAST or Polar) to observe electron dispersion. Another part of the problem is that simultaneous wave and particle observations have not been available. Tanaka et al. (2005a), see Fig. 10, showed sounding rocket data with sufficient time resolution to observe time dispersed features. Although they did not show simultaneous wave observations, in a companion paper Tanaka et al. (2005b) used a simulation technique to show that the observed dispersion signatures could be reproduced by a propagating Alfvén wave.

It has been similarly difficult to link Alfvén waves with ion outflow on a regular basis. Chaston et al. (2015) performed a statistical study of Alfvén waves that included the dayside cusp but at somewhat lower latitudes, finding enhanced Alfvén waves and ion outflow during geomagnetic storm main phases. This study inspired Hatch et al. (2016) to extend it to higher latitudes using FAST data, finding enhanced Alfvén wave occurrence and associated ion fluxes in the cusp during storm main phases. Strangeway et al. (2005) derived scaling laws for the correlation between DC Poynting flux and electron precipitation to ion outflows. Brambles et al. (2011) extended this regression analysis to include AC (Alfvénic) Poynting flux.

At 590 km altitude, TRACERS is well-positioned to detect Alfvén waves and their associated particle signatures. Alfvén waves are identified by their electric and magnetic field signatures. The physics of these dynamic features define driving requirements for the electric, magnetic, and search coil instruments, e.g. electric and magnetic field measurements must determine propagation directions up to several hundred Hz to separate Alfvén waves and other wave modes such as electromagnetic ion cyclotron (EMIC) waves (which have very different propagation characteristics). The DC magnetometer must measure up to a few Hz to separately identify field-aligned currents. Rapid electron spectra with sweep rates of the order of 50-100 ms are needed to resolve time-dispersed electrons. The effects of Alfvén waves are identified in the electrons and the resulting ion outflow is measured by the 180° pitch angle field of view of the ion instrument.

### Science Objective 3 Science Closure

**Fig. 11:**
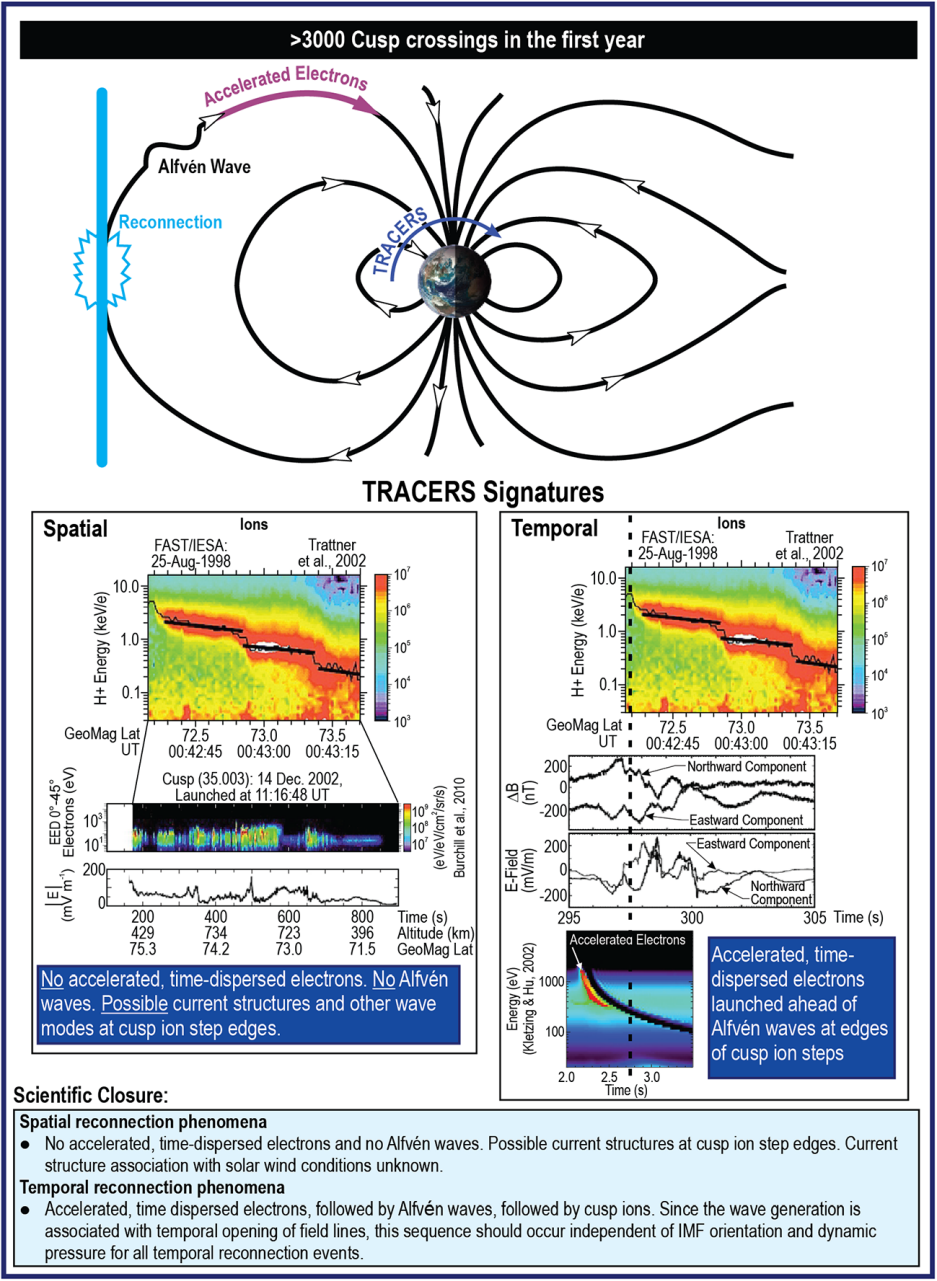
Science Objective 3 - determine to what extent dynamic structures in the cusp are associated with temporal versus spatial reconnection

Science closure of Objective 3 is achieved by determining the relationship between Alfvén waves, precipitating electrons, and ion outflow. If reconnection at the magnetopause is temporally variable, then this process should launch Alfvén waves toward the ionosphere as illustrated in Fig. 11. Conversely, steady-state reconnection from different locations would not be expected to be associated with Alfvénic activity because there is no temporal variation that would launch discrete wave signatures. However, at the boundaries of the cusp ion steps (and possibly electron steps as well) there could be both current structures associated with spatial boundaries and particle signatures or unstable wave modes associated with energy and particle release in reconnection. The fact that Alfvén waves occur essentially 100% of the time somewhere in the cusp (Fig. 9) may be because reconnection is either spatially or temporally variable essentially all the time, and TRACERS will provide the ground truth to determine if this is the case.

By separating spatial and temporal cusp ion signatures, TRACERS will confirm that spatial and temporal events have different wave and particle signatures as illustrated in Fig. 11. For spatial events, no accelerated, time-dispersed electrons or Alfvén waves are expected. Ion outflow occurs within the cusp but not necessarily associated with waves. For temporal events, time dispersed electrons are followed by Alfvén waves (and perhaps other waves) and ionospheric ion outflow. These wave and particle signatures should be independent of solar wind dynamic pressure. TRACERS has the requisite measurements over a wide range of solar wind conditions, along with the needed simulations, to reconcile electron burst observations (dispersed or otherwise), to directly connect Alfvén wave fields to the particle events and to associate these features with ion outflow, providing another aspect of discovery science.

Completing this connection between cusp phenomena and spatial/temporal reconnection may enable existing ground asses to inexpensively and comprehensively monitor the nature of reconnection and plasma transfer into the magnetosphere. For example, all sky cameras measure poleward moving auroral forms (PMAF) and incoherent scatter radars measure cusp boundary motions. If these are associated with temporal reconnection variability and precipitating electrons, as suggested by Lockwood et al. (2005), then based on TRACERS results these existing, and relatively inexpensive assets could monitor reconnection rate variability over a broad range of local times and over sustained periods of time via a new understanding of what can be inferred from their measurements.

### Modeling TRACERS Cusp Observations for Global and Local Context

Spatial reconnection structures at the magnetopause are often 3-D in nature so the corresponding ion signatures in the cusp may exhibit temporal effects as suggested by Trattner et al. (2002a), as well as local and global effects. Hybrid simulations will be used to support the interpretation of the TRACERS observations, investigate the implications of these results on magnetospheric dynamics, understand the ion kinetic physics in the cusp, and study the waves and other electrodynamics associated with cusp reconnection signatures. These simulations provide important context for understanding the interconnection of all three science objectives and ensuring full science closure for the TRACERS mission. TRACERS treats the modelling component as an additional instrument to complement the physical instrument suite.

**Fig. 12:**
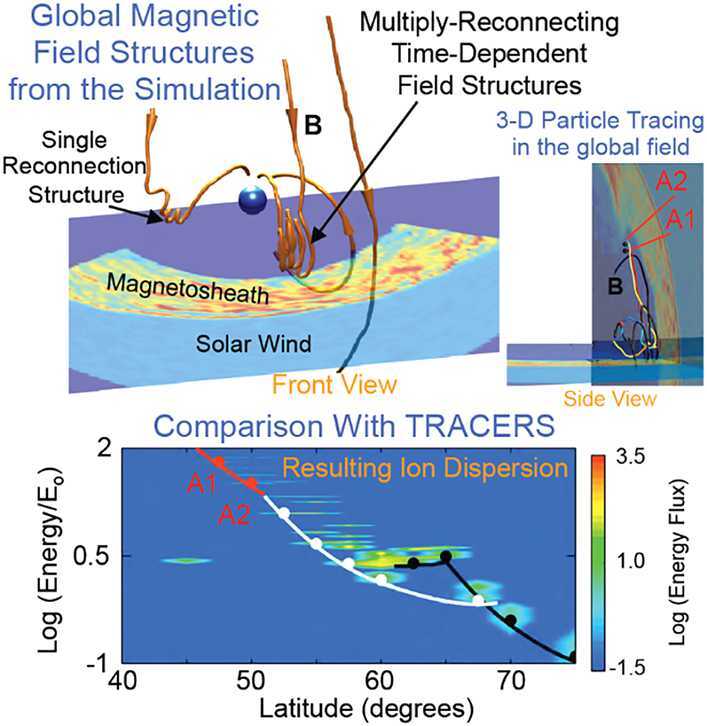
Results of a global hybrid simulation of the cusp (Tan et al. 2012). (Upper panels) The global reconnecting field lines at the magnetopause and particle tracing. (Lower panel) the resulting cusp ion dispersion predicted from the particle tracing. These simulations are conducted for a select number of TRACERS cusp encounters to determine how the 3-D structure of the reconnecting magnetic field in the top panel relates to the modeled cusp ion dispersion in the bottom panel. The bottom panel is compared to the ion dispersion observed by TRACERS

To complete Science Objective 1, the spatial structure and temporal evolution of the magnetopause reconnection (Lin 2001; Tan et al. 2011) will be examined in the global simulations, and the output of these simulations, the modeled cusp ion signatures, will be compared directly with TRACERS observations. Figure 12 shows examples of simulation results (Tan et al. 2012) and illustrates what will be compared with TRACERS observations. In this simulation, multiple X-line reconnection events are present at different latitudes of the magnetopause (top left panel). A stepped energy spectrum of the field aligned precipitating magnetosheath ions is observed in the cusp (bottom panel, at r = 7 RE, but the same dispersion at lower altitudes). Tracing particle trajectories in the simulation (top right panel) associates the ion energy spectrum with multiple steps (bottom panel) with the multiple reconnection events at the magnetopause The dispersive low energy cutoff signature is generally consistent with that in Lockwood et al. (1994).

According to Trattner et al. (2002a), spatial events may also contain temporal features in the cusp ion spectrum. Moreover, the particle acceleration does not occur at a single point in space and time as in simplified models but is a much more involved process. The trends in typical signatures obtained from the simulations under various solar wind conditions will inform the interpretation of the observational results. Combined with the TRACERS observations, these detailed simulations provide the connection between pulsed reconnection and bursty magnetopause flux transfer events and will demonstrate the broader implications for magnetospheric dynamics. Confidence in the simulations will be realized by showing that they reproduce observed ion dispersions from both TRACERS spacecraft.

To complete Science Objective 2, the reconnection rate evolution in the simulation is estimated by determining the reconnection electric field around the X-lines over the magnetopause. The reconnection X-lines are identified in the global simulation as described by Tan et al. (2011). The roles of variable reconnection rate on the particle transport will be elucidated by examining simulations with quasi-steady and time-dependent reconnection at the magnetopause. Furthermore, these detailed simulations provide the direct connection between the reconnection rate variability observed by TRACERS and magnetopause flux transfer events in the simulation and demonstrate the broader implications for magnetospheric dynamics.

To complete Science Objective 3, the physics of Alfvén waves associated with quasi-steady and transient magnetic reconnection will be simulated. Alfvénic waves and Kinetic Alfvén Waves will be identified as in previous simulations of a local reconnection layer (Liang et al. 2016; Lin and Xie 1997), and the parallel Poynting flux mapped to the TRACERS altitude (Zhang et al. 2014). The generation and propagation of Alfvén waves will be examined in the global simulations and in detail using additional local scale “slab” hybrid simulations (Lin and Swift 1996; Lin and Xie 1997). Test particle traces provide insight on Alfvén wave acceleration. In addition to reconnection, solar wind dynamic pressure variations and foreshock compressional waves, likely to be associated with the quasi-parallel shock, may also interact with the magnetopause and generate Alfvénic waves (Lin et al. 2010, 2012; Shi et al. 2013).

The IMF cone angle will determine whether the cusp is connected to the quasi-parallel bow shock (e.g., Trattner et al. 2001). The Alfvénic signatures in these processes are expected to be different from those associated with reconnection, which is accompanied by ions crossing an open magnetopause. Similarly, Alfvénic signatures from Kelvin Helmholtz waves at the magnetopause (which occur more often for northward IMF conditions) should be different. These simulations, and the observed Alfvén waves by TRACERS, will determine if the generation mechanisms are due to reconnection or other processes.

### Science Summary

Science Objective 1 will use a large database of cusp crossings to determine if the magnetopause reconnection process is primarily temporally sporadic from a fixed spatial location or temporally steady from a variety of spatial locations. Science Objective 2 will use the temporally variable events to quantify the variability in the reconnection rate. Two independent methods (Fig. 7) will be used to determine the reconnection rate variability and, for a select number of events, global modeling will provide a measure of the absolute change in the reconnection rate. Science Objectives 1 and 2 are linked with the Decadal Survey goal to discover how magnetic reconnection is modulated (and to a similar link to a Heliophysics Roadmap goal). In Science Objective 3, cusp dynamic structures are associated with temporal/spatial reconnection. The combination of global modeling, quasi-local modeling, and constraints on the structures in the cusp from the observations provide the magnetopause’s global dynamic structure. The combination of observations and modeling to achieve Science Objectives 1 and 2 links with both Decadal Survey and Heliophysics Roadmap goals to understand how plasma interacts within the magnetosphere and at its boundaries. Science Objectives 1, 2, and 3 will achieve the overall TRACERS science goal and are accomplished with carefully tailored, identical instrument sets on the two spacecraft.

### Driving Science Requirements

All three science objectives drive important measurement requirements that determine the size, mass, power, and telemetry of the TRACERS instruments. The driving requirements for the instruments are summarized in Table 1.

**Table 1:** Driving requirements for the TRACERS instruments and their associated science objectives

Instrument	Driving Science Requirement and Science Objective (SO)	Instrument Requirement
ACE and ACI	Measure the full range of cusp ion and electron precipitation (Science Objectives 1 and 2)	Geometric Factors and Instrument Size
ACE and ACI	Measure the full range of ion and electron precipitation from low energy cusp precipitation to ring current energies (Science Objectives 1 and 2)	Energy Range
ACI	Measure possible energy changes within a cusp ion step (Science Objective 2)	Time Resolution
ACE	Measure electron dispersion at the leading edge of the cusp (Science Objective 3)	Time Resolution
EFI	Measure perpendicular electric field to determine cusp convection velocities (Science Objectives 1 and 2)	Time Resolution and Accuracy
EFI and MSC	Measure AC electric and magnetic fields to identify Alfvén and other waves (Science Objective 3)	Frequency Range and Resolution
MAG	Measure DC magnetic field to identify field-aligned currents in the cusp (Science Objective 3)	Time Resolution and Accuracy

The TRACERS orbit is fixed in the inertial frame, whereas the location of the Cusp is essentially constant in the Solar Magnetic frame. The relation between these frames varies as the Earth’s dipole rotates daily. Consequently, the TRACERS orbit transits East or West of the expected cusp location a significant percentage of the time. Further, the shape and extent of the Cusp varies with solar wind parameters, which can make it a smaller target. Taken together, this results in >3000 predicted cusp crossings out of nearly 5500 orbits in the twelve month baseline mission. The importance of IMF orientation on spatial or temporal reconnection variability (Science Objective 1) will be answered with high accuracy due to having >100 events in each IMF orientation category. Assuming temporal events constitute 50% of the total events, Science Objective 2 will have a statistically significant number (>100) of events to determine if there are large variations in reconnection rate for nominal solar wind dynamic pressures. However, a statistically significant number of separations over the full range from 10 s to 2 min and over all dynamic pressures requires the full twelve months of operations. For Science Objective 3, the first 6 months will provide an adequate database of events to identify Alfvénic signatures and electrodynamic variability in the cusp ionosphere; however, events will be gathered throughout the 12 month baseline mission.

## Science Instruments

**Fig. 13:**
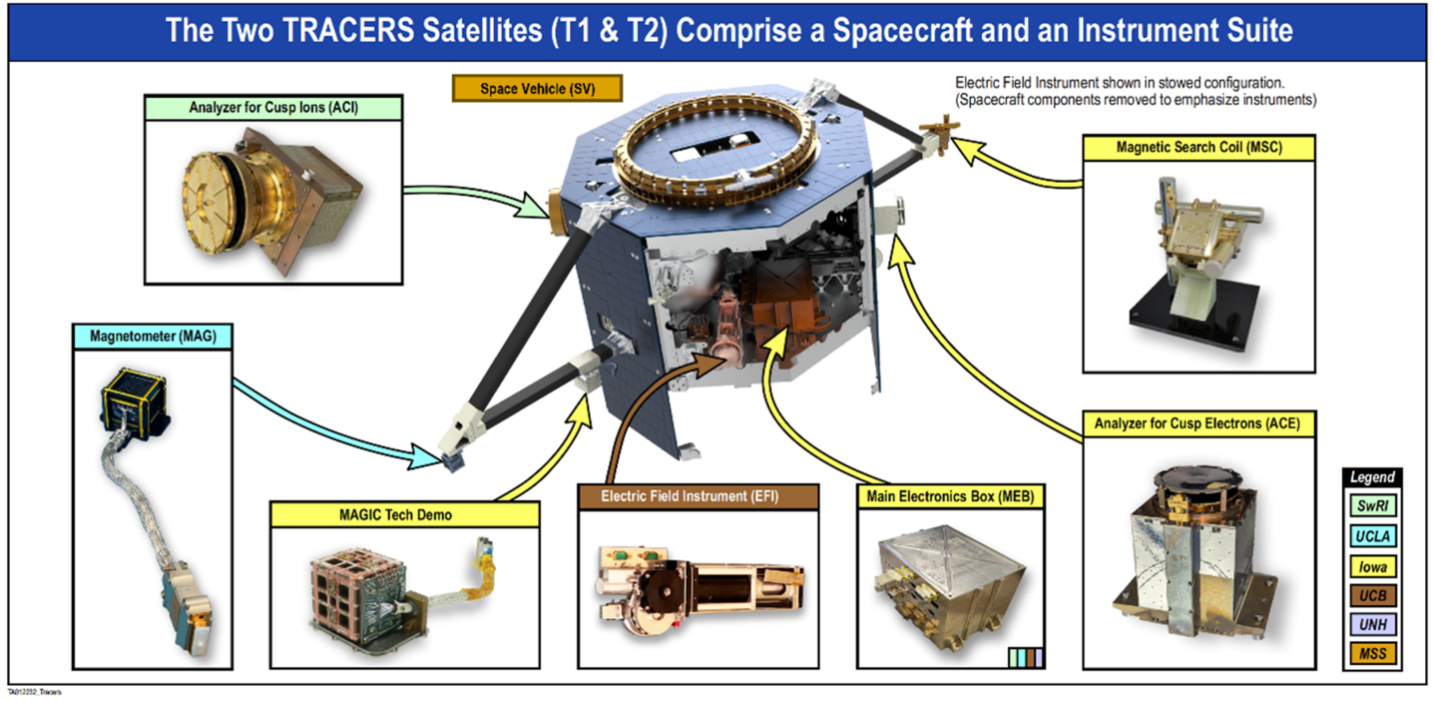
The TRACERS Instrument Suite is identical on both spacecraft

The TRACERS instruments are chosen specifically to achieve the science objectives (SOs), requiring a combination of accurate and sensitive charged particle and field measurements with high temporal resolution and two-point measurement capability to distinguish between spatial and temporal variability. TRACERS utilizes identical integrated instrument suites (Fig. 13) on two spacecraft to achieve the science objectives.

The TRACERS instruments provide a robust set of observations tightly coupled to the science objectives. Each spacecraft carries an Analyzer for Cusp Ions (ACI) to measure ion energy-pitch angle distributions every 0.31 s, an Analyzer for Cusp Electrons (ACE) to measure electron energy-pitch angle distributions every 50 ms, a 3-axis fluxgate Magnetometer (MAG) to measure the background field, currents, and low frequency waves at 128 samples/s, a 2-axis Electric Field Instrument (EFI) to determine the perpendicular electric field at 128 samples/s (used to compute the $E\times B$ flow velocity) and measure higher frequency waves, a 3-axis Magnetic Search Coil (MSC) to measure magnetic field fluctuations at 2048 samples/s, and a common Main Electronics Box (MEB). The MAGnetometers for Innovation and Capability (MAGIC) instrument is a technology demonstration hosted payload and is not required for TRACERS science closure. The MAG, MSC, and MAGIC sensors are isolated from the magnetic noise of the spacecraft using rigid brackets constructed from rectangular cross-section carbon fiber composite tubes. The tube that accommodates the magnetometer sensors mounts radially from a spacecraft face sheet as show in Fig. 13. The TRACERS Instrument Suite (TIS) is composed entirely of heritage sensors optimized for the low-altitude cusp, each of which has previously successfully flown and operated in comparable environments.

**Fig. 14:**
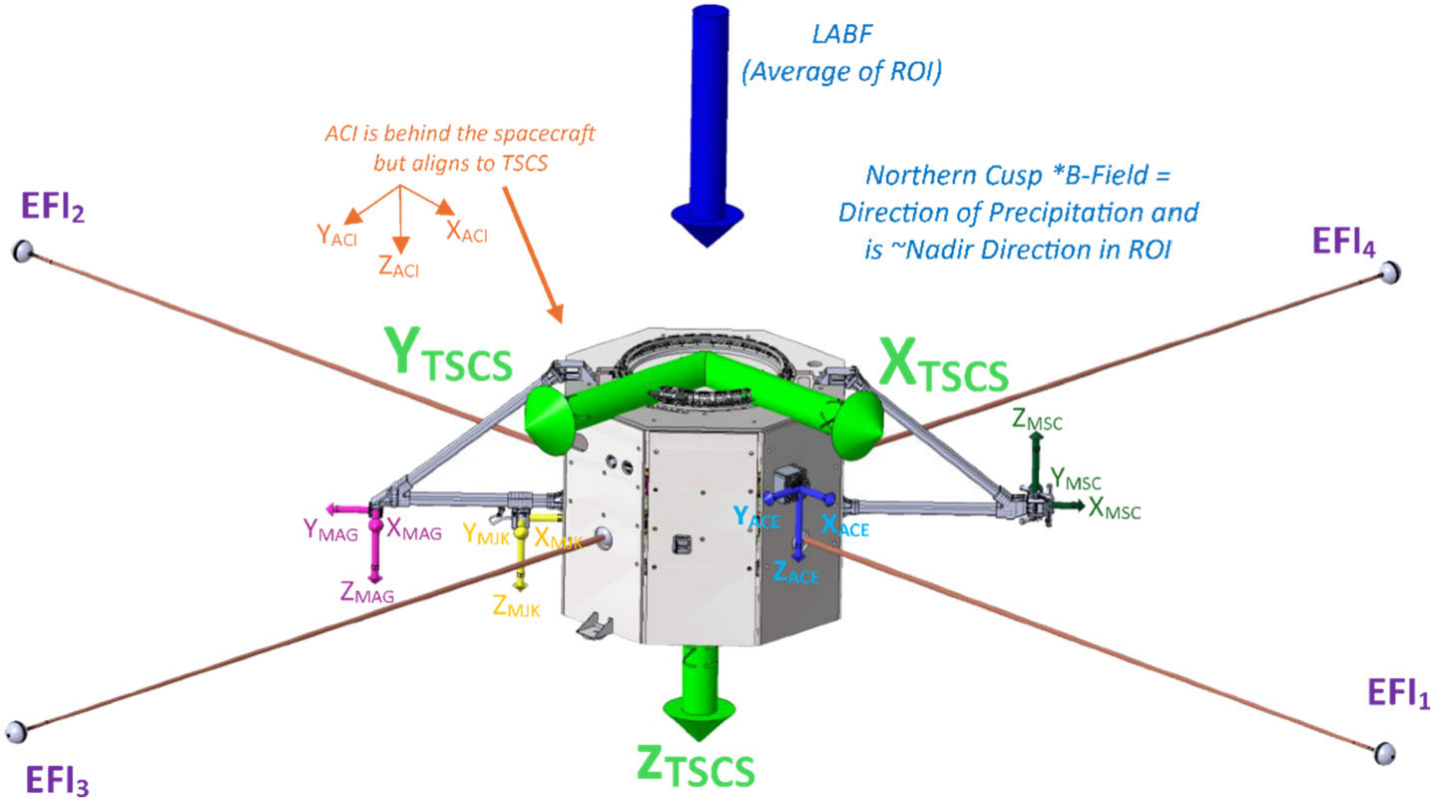
Instrument orientation during the Region of Interest (ROI). Note that the 2D electric field measurement is perpendicular to the background field and both ACI and ACE can instantaneously resolve complete pitch angle distributions

Each TRACERS spacecraft is oriented such that (Fig. 14) during the ROI (the northern magnetospheric cusp), both spacecraft spin axis are aligned with the background magnetic field (B).Both vehicles are deployed and operate spinning counterclockwise about their Z axis. The particle instruments, ACI and ACE, mount on outside face sheets such that they can resolve the full pitch-angle distribution. The EFI Stacer booms deploy into the spin plane so that the perpendicular electric field is fully resolved while in the ROI.

### Analyzer for Cusp Ions (ACI)

Southwest Research Institute provided TRACERS with one Analyzer for Cusp Ions (ACI) per spacecraft to measure the 3-D ion velocity distribution (Fuselier et al. 2025, this collection). Each ACI is a toroidal top-hat electrostatic analyzer (ESA) that measures cusp ion energy dispersion signatures critical for distinguishing temporal versus spatial reconnection variability. ACI provides the needed sensitivity and energy and time resolution to resolve these signatures and achieve the TRACERS Science Objectives.

Each ACI consists of an ESA with microchannel plate (MCP) detectors that have heritage from MMS, TRICE-2, and CuSP. ACI has a 360° $\times \ \sim $6° field of view oriented tangent to the bus panel and containing the spin axis. Therefore, ACI measures two complete energy-angle distributions per energy sweep in the center of the ROI. This measurement meets the driving time resolution requirement to unambiguously determine the low energy cutoff of cusp ion steps to achieve the science objectives within a single cusp ion step. ACI has a 360° collimator, through which ions enter the ESA, where a voltage between the two concentric toroids selects ion energy. The ESA optics have been tested extensively in the development of the MMS Hot Plasma Composition Analyzer (HPCA) (Young et al. 2016).

### Analyzer for Cusp Electrons (ACE)

The University of Iowa provided TRACERS with one ACE per spacecraft to measure the 3-D electron velocity distribution (Halekas et al. 2025, this collection). ACE utilizes a heritage ESA to provide measurements of cusp and magnetospheric electrons at high cadence, with high energy and angle resolution. These capabilities enable identification of cusp dynamic structures and the determination of open-closed field line boundaries with sub-kilometer spatial resolution. ACE provides the needed sensitivity and energy, angle, and time resolution to locate boundaries and measure dispersed electron signatures to achieve the TRACERS Science Objectives.

ACE is nearly identical to those designed and successfully flown by the University of Iowa on several rocket flights, including the ACES and CHARM-2 missions, RACE, HiBAR, TRICE-2, and ACES-II. These heritage sensors have already flown successfully to the TRACERS altitude and returned excellent science data.

ACE utilizes a similar design to ACI, based on the original Carlson et al. (1982) concept. ACE biases the inner of two concentric hemispheres to a positive voltage to select electrons by energy, with high energy and angular resolution ensured by the natural ESA focal properties. ACE is an imaging sensor that counts single electrons, using the following components: a chevron pair of MCPs located below the exit of the ESA to produce charge pulses with an amplitude of $\sim 10^{6}$ electrons; a segmented anode to collect these charge pulses; and Amptek A121 charge-sensitive amplifiers to convert these pulses into digital counts. By sweeping the hemisphere bias voltage, ACE very rapidly (50 ms) covers the full energy range for all look angles. ACE’s symmetry axis is aligned perpendicular to the spin axis (Fig. 14), so that its 210° × 7° field of view instantaneously covers all angles from the spacecraft axis, ensuring that ACE always covers all electron pitch angles at least once per spin.

### Electric Field Instrument (EFI)

The Space Sciences Laboratory at the University of California Berkeley provided TRACERS with one EFI group (4-sensor quartet) per spacecraft, which makes a 2-D measurement of the in situ electric field, using the double probe technique (Bonnell et al. 2025, this collection). The current biased double probe E-field measurement technique has heritage going back over 40 years (Maynard 1998; Pedersen et al. 1998). While other methods have been used to derive the electric field from plasma drift (ion drift meters, electron drift instruments), or different electrode configurations (whips, wire antennas), the design simplicity, high degree of symmetry, and low levels of systematic error under illumination or in drifting plasmas make the double probe technique appropriate for TRACERS.

The use of Stacer booms to deploy the E-field sensors away from the body of the spacecraft and provide for sensor separation dates back almost as far, with University of California Berkeley’s Space Science Laboratory a leader in the original development and continuing use of such booms. The TRACERS boom design has flown on dozens of sounding rockets, as well as on DEMETER, in similar or identical plasma conditions (Berthelier et al. 2006). The DCLF EFI preamps (sphere mounted) are derived from the OP-15-based, single op-amp designs flown on THEMIS, Van Allen Probes, GREECE, and TRICE-2. The HF EFI preamps (body-mounted) are derived from the AD8001S design flown on Parker Solar Probe (PSP). The sensor biasing and power supply Boom Electronics Board (BEB), is a simplified version of the PSP-Fields Antenna Electronics Board, which in turn has heritage in BEBs flown on THEMIS-EFI, Van Allen Probes EFW, and POLAR-EFI. The analog and digital waveform signal processing board (EFI Signal Processing [ESP]) is derived from the THEMIS-DFB and the GREECE processing board. EFI provides the needed sensitivity, precision, and time resolution to resolve DC and AC electric fields to achieve the TRACERS Science Objectives.

### Fluxgate Magnetometer (MAG)

The University of California at Los Angeles provided TRACERS with one MAG sensor per spacecraft to measure the vector magnetic field (Strangeway et al. 2025, this collection). The MAG sensor is mounted on a rigid carbon-composite fixed bracket to isolate it from AC and DC stray magnetic fields. MAG provides the needed sensitivity, precision, and time resolution to resolve DC and low frequency AC magnetic fields and achieve the TRACERS Science Objectives.

The TRACERS MAG is based on the high heritage design of magnetometers flown by University of California Los Angeles starting in the Apollo era. Missions that have flown University of California Los Angeles magnetometers include the International Sun Earth Explorers (ISEE-1 to -3) in the 1970s and 1980s, GGS Polar and FAST in the 1990s, Space Technology 5 (ST5), MMS, and the Mars lander mission, InSight. The TRACERS MAG has heritage from the InSight design. The InSight magnetometer sensor design is essentially the same as the MMS magnetometer sensors.

The magnetometer internally samples data at 128 samples/s, with 24-bit resolution. The MEB down-samples this to the desired data rate and bit resolution. The dynamic range of the TRACERS sensor is ±60,000 nT, a factor of three increase over the InSight sensor. MAG provides the needed sensitivity, precision, and time resolution to resolve DC and low frequency magnetic electric fields to achieve the TRACERS Science Objectives.

### Magnetic Search Coil (MSC)

The University of Iowa provided TRACERS with one MSC per spacecraft (Hospodarsky et al. 2025, this collection), each consisting of three identical search coil sensors mounted in a tri-axial configuration with corresponding preamplifiers. The MSC is mounted on a fixed rigid bracket with the same design and test flow as the MAG boom.

Two sensors are oriented parallel to the two electric dipole antennas and the third parallel to the spacecraft spin axis. The TRACERS MSC is based on previous search coil sensors designed and built at University of Iowa, including the ISEE-3, GGS Wind, Juno, and Van Allen Probes search coils. To improve low frequency sensitivity, each MSC sensor unit contains two coils of 26,000 turns of #44 copper wire, resulting in a useable frequency range of ∼1 Hz to a few kHz. Each sensor unit utilizes a 15 cm long mu-metal core (same as Juno). The MSC preamplifiers are based on Van Allen Probes (Kletzing et al. 2013) and Juno (Kurth et al. 2017) designs and incorporate a flux-feedback design that flattens the response near the coil resonance so that it is more easily calibrated. The MSC sensors and preamplifiers are designed to operate within a wide temperature range, with a thermal design not requiring heaters.

The analog signals from the MSC preamplifiers are converted to digitized waveforms of 16-bit samples at 2048 samples/s by ADCs on the EFI board. The full waveforms are sent to the ground for further processing. The MSC runs continuously throughout the orbit (avoiding power cycling and operational complexity), with the Common Data Processing Unit (CDPU) heavily decimating the data, outside of the ROI. MSC provides the needed sensitivity, precision, and time resolution to resolve AC magnetic fields and achieve the TRACERS Science Objectives.

### Main Electronics Box (MEB)

**Fig. 15:**
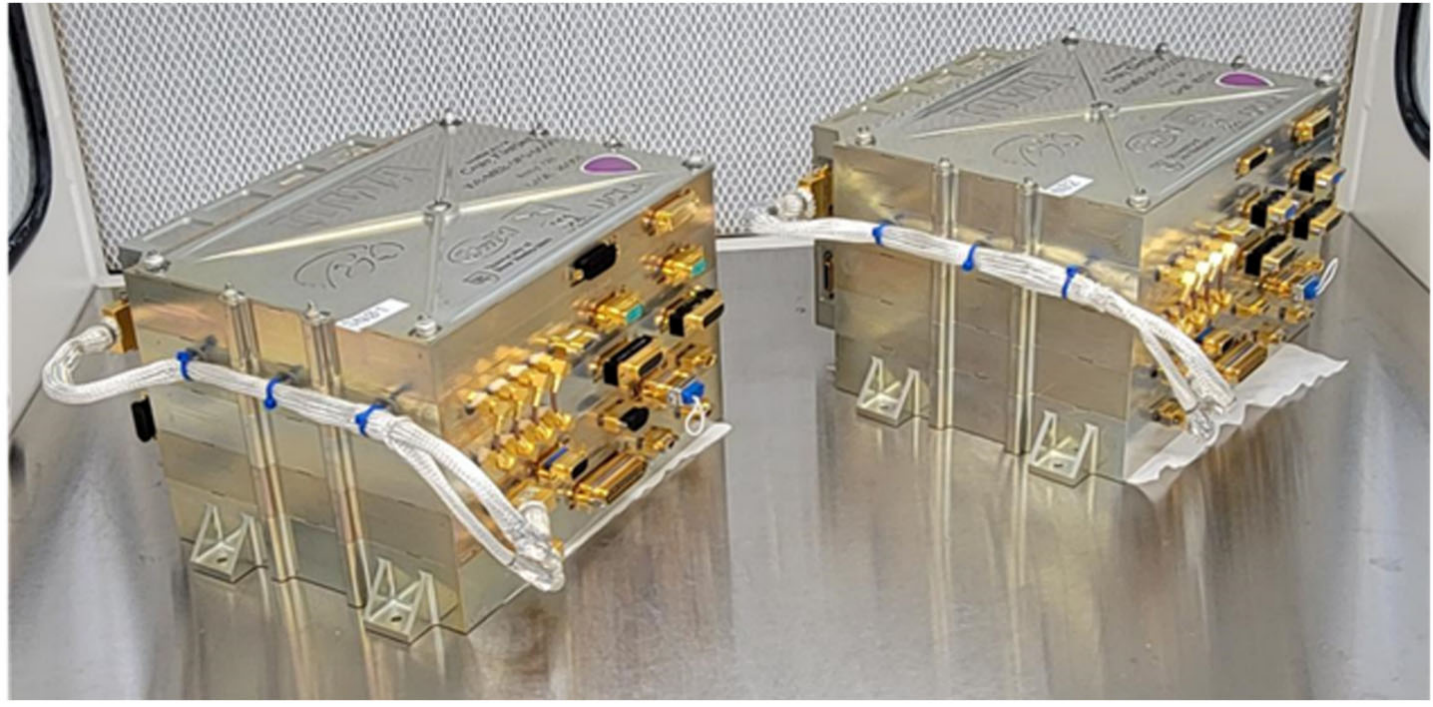
Main Electronics Box (MEB) flight hardware

The MEB (Fig. 15), with high heritage from the Van Allen Probes EMFISIS experiment, provides a unified power, telemetry, and command interface between the TIS and the spacecraft. All electronics except for the ACE and ACI sensor electronics are integrated in the MEB, which consists of a stack of six 6” × 9” boards: the Low Voltage Power Supply (LVPS), the CDPU, the two EFI boards, and the MAG boards. Each board is integrated in a frame that provides a mounting fixture for any needed external connections, and a back plane carries power and signals between the five boards in the MEB, like the Van Allen Probes design. The MAGIC technology demonstration has a separate small electronics box mounted to the side of the MEB stack. A dedicated and switched main voltage services allows the technology demonstration to be shut off if it violates the do-no-harm tenants of its accommodation.

The LVPS, with hardware based directly on the Van Allen Probes EMFISIS design, converts primary unregulated power from the spacecraft to regulated secondary digital and analog voltages to run the CDPU and the fields (MAG, MSC, and EFI) electronics. The LVPS produces the supply voltages required by the CDPU and for MAG, MSC, and EFI. The use of the Van Allen Probes design, which introduced no measurable interference to very similar measurements on the EMFISIS experiment, ensures that TRACERS makes clean waves measurements.

### Central Data Processing Unit (CDPU)

**Fig. 16:**
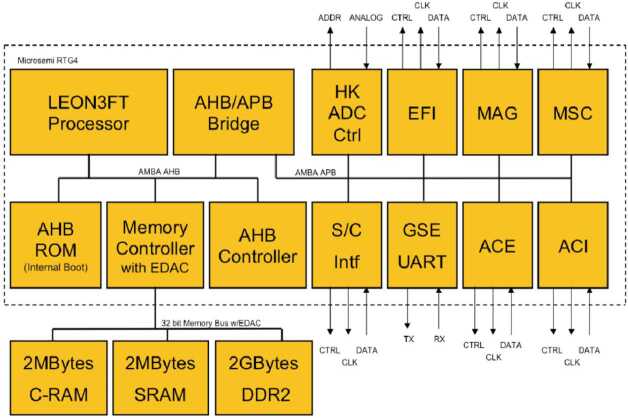
Central Data Processing Unit (CDPU) lock diagram

The Central Data Processing Unit (CDPU) shown in Fig. 16 has both hardware and software based directly on the Van Allen Probes EMFISIS design, receives power from the LVPS and logically interfaces to each of the instruments via Low Voltage Differential Signaling (LVDS) serial communication links. The CDPU controls the sensors, receives data from the sensors and packetizes it, and communicates with the spacecraft processor. To simplify the ACE outboard electronics, the CDPU includes sequencer tables to control stepping of high voltage and the accumulation intervals. The ACI and EFI interfaces are highspeed serial command, clock, and data signals. The MAG interface is drawn directly from the same instrument electronics on MMS FIELDS. The use of LVDS ensures low ground currents and maximizes power isolation.

The CDPU is built upon the LEON3FT core implementation of a SPARC processor. The LEON3FT core is in a Microsemi RTG4 Field-programmable gate array (FPGA), which is radiation tolerant, latch-up immune, and Triple Modular Redundant (TMR). The basic system architecture is based on processor designs on both Van Allen Probes EMFISIS and MMS FIELDS.

The CDPU contains non-volatile C-RAM (chalcogenide RAM) for operating code and table storage, Static Random Access Memory (SRAM) for temporary and program execution, and 512 MB of Double Data Rate Synchronous Dynamic Random-Access Memory (DDR2 SDRAM) to store telemetry data for later transfer to the spacecraft computer. Basic boot software is contained in the RTG4, providing rudimentary commanding for system initialization and fault recovery. The operating code is based on the Real-Time Executive for Multiprocessor Systems (RTEMS) kernel and written in C.

The spacecraft interface uses LVDS serial command, clock and telemetry. The CDPU logically decodes commands for reset with vectored booting, C-RAM write protect, and watchdog override. Other commands are placed in a FIFO for processor decoding. The telemetry is placed in a First In First Out (FIFO) buffer by the processor and transferred to the spacecraft via a Finite State Machine (FSM).

The flight software design uses a model based on heritage from Van Allen Probes EMFISIS and MMS FIELDS instruments, which interfaced with very similar instrumentation with comparable or higher data throughput and processing requirements.

### TRACERS Modeling Integration

Global hybrid simulations with the ANGIE3D code (Lin et al. 2017) will be conducted on a select number of cusp crossings to elucidate the global 3-D structure of magnetopause reconnection and the associated observable effects in the cusp (Lin et al. 2025). Simulations of reconnection in the local magnetopause boundary layer will be conducted correspondingly to zoom in on the detailed reconnection physics. These combined simulations provide understanding of the global transport resulting from magnetopause reconnection as a function of solar wind conditions. The ion acceleration, heating, and velocity distributions will be examined to understand the signatures of ion cusp precipitation. The particle trajectories will be traced self-consistently in the global simulation to determine the origin of the ions in a typical cusp spectrum (Tan et al. 2012).

### MAGnetometers for Innovation and Capability (MAGIC)

MAGnetometers for Innovation and Capability (MAGIC) is a technology demonstration payload (Miles et al. 2025) for new fluxgate magnetometer technology (Miles 2017; Miles et al. 2017b,a, 2019; Narod and Miles 2024). The Do-No-Harm technology demonstration flight provides flight-heritage to the design through on-orbit cross-comparison with the MAG payload. Additionally, having two magnetometers potentially allows additional mitigation of the spacecraft field.

Each MAGIC payload comprises a fluxgate sensor, mounted on the same bracket as MAG, and a dedicated electronics box mounted to the side of the TRACERS Main Electronics Box (MEB). MAGIC will demonstrate two types of fluxgate sensor, a traditional 1” ringcore sensor using new S1000-compatible fluxgate cores and the new ‘Tesseract’ sensor (Greene et al. 2022) based on new race-track geometry cores. MAGIC inherits its data processing and calibration software (Broadfoot et al. 2022) from the MGF (Wallis et al. 2015) instrument on the e-POP spacecraft (Yau and James 2015). MAGIC does not formally contribute to any of TRACERS’ science objectives since it is an NPR 7120.8A technology development payload.

### Data Sufficiency

TRACERS will encounter the northern cusp >3000 times during its one-year primary mission. TRACERS downloads high-rate data from each passage across the ROI, returning comprehensive measurements for every one of these cusp crossings. All instruments are operated at full sample rates during passage through the ROI, providing the highest possible resolution of all measured quantities and providing all the needed quantities for science closure.

As discussed in detail in Sect. 3.11, TRACERS provides statistically significant sampling over a full range of IMF geometries and solar wind dynamic pressure conditions. This provides an abundance of data for closure of Science Objectives 1 and 2. The near alignment of the TRACERS spin axis to the background magnetic field in the ROI provides excellent coverage of electron and ion pitch angles, aiding in closure of Science Objective 3.

### Instrument Operations

TRACERS utilizes a simple operational scheme, with the MEB providing the power, command, and data interface between the instruments and the spacecraft. The spacecraft monitors the primary currents for the TIS power services [CDPU; Fields (MAG MSC, EFI); ACE; ACI; MAGIC] and switches them off if overcurrent conditions occur. The CDPU also monitors instrument housekeeping and triggers fault protection actions by the MEB and/or spacecraft if any monitor values exceed predefined limits. The CDPU performs digital processing tasks, including binning and packetizing data from the sensors, and compressing and transferring packets to the spacecraft for telemetering.

The CDPU can execute individual commands or groups of commands. All commands were tested in a testbed (“FlatSat”) constructed from flight-like EM and/or spare units, with simulator boards in place of sensor elements. This testbed operated like the flight suite from an interface and processing perspective. All scripts were thoroughly tested on the testbed, then approved by relevant instrument, operations, and spacecraft personnel, before upload to the spacecraft. Check-summing of all commands and lookup tables on-board guards against ill-formed or corrupted commands.

### Operational Modes

Each sensor operates continuously in a single mode, until commanded by the CDPU, which can change the sensor operational parameters and telemetry rates as needed by changing appropriate values in lookup tables and/or switching between multiple preloaded tables. During nominal operation (science main mode), each sensor has two modes per orbit: one utilized in the ROI and one in the remainder of the orbit. Additional modes are utilized for checkout and periodic calibration activities.

Most of the science data are collected during the cusp encounters. The CDPU can decimate instrument telemetry to return a low rate of data for trending and calibration purposes from the remainder of the orbit. The CDPU includes internal memory (2 GB) to store high-rate data from the ROI (with storage for up to 48 cusp encounters), allowing storage of multiple days of data onboard so that, in the event of missed contacts, no data are lost.

### Instrument Accommodation

The TRACERS architecture simplifies spacecraft accommodation, with the internally mounted MEB providing the power, telemetry, and control interface to the spacecraft. The MSC, MAG, and MAGIC sensors are accommodated on 70 cm fixed brackets. The EFI sensors are deployed on 3-m Stacer booms provided by University of California Berkeley Space Science Laboratory. The ACE and ACI sensors are deck-mounted (partially recessed to ensure clearance in the fairing), with their symmetry axes perpendicular to the spin axis, so that they view all angles from the spacecraft spin axis instantaneously, ensuring near-complete instantaneous pitch angle coverage in the ROI. ACI views the entire angular range from 0° to 180° from the spin axis in 2 directions simultaneously, enabling redundant coverage of most ion pitch angles. Since very low energy (<10 eV) ions are not required to achieve TRACERS science objectives, the ACI field of view can include electric field Stacers and magnetic field booms. ACI is mounted so that its field of view for the critical downward-going ions is unobstructed. ACE is mounted close to a corner so that its 210° × 7° field of view looks out from the spacecraft, without any booms or Stacers in its field of view, so that photoelectrons and/or secondary electrons from spacecraft or instrument surfaces cannot contaminate measurements.

## Science Orbit and Observation Coverage

**Fig. 17:**
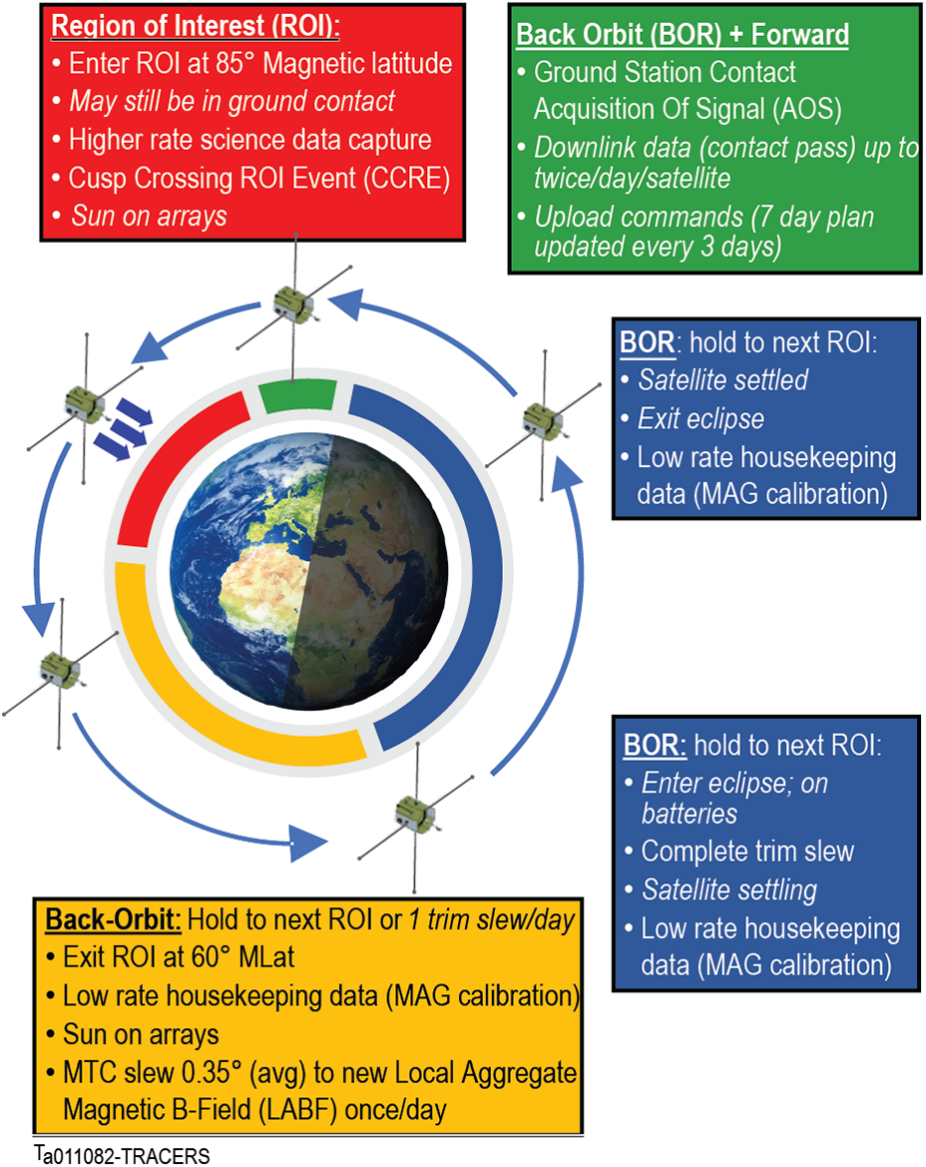
Orbit-in-the-life Design Reference Mission Scenario

TRACERS has two nominal science data states (Fig. 17), the Region of Interest (ROI) which is entered at 85° and exited at 60° magnetic latitude, and the Back Orbit (BOR). The instruments, including ACE/ACI HV, stay in one constant “on” state during both ROI and BOR, requiring no instrument state changes – though data rates do change.

### Region of Interest (ROI)

The CDPU records full-rate (594 kbps, uncompressed for <7 min) science data, via a table-driven filter, upon entering the ROI to measure the cusp. The CDPU in the MEB packetizes the science and housekeeping data into separate Consultative Committee for Space Data Systems (CCSDS) Virtual Channel Data Units (VCDUs) and stores them in SRAM, for later parsing by the MOC and SOC. TRACERS maintains a fixed Sun-positive inertial attitude, aligned to one day’s local aggregate magnetic B-Field vector, using attitude table values uploaded every 3 days from the SOC, covering a 7-day operational period.

### Back Orbit Typical

As the TRACERS spacecraft exits the ROI into the BOR it holds attitude for the next ROI while the low-rate filter table decimates science data (principally retaining low-rate background data) with nominal rate housekeeping data (13 kbps, low rate) acquisition. The MAG and EFI collect low-rate data in the BOR to support their on-orbit B- and E-field calibration algorithms. ACE/ACI gain-stepping long-term calibrations are performed at a monthly cadence, briefly in the BOR.

### Slew

Once a day, at BOR onset, each spacecraft initiates a minor (0.35° average) trim slew to the next Local Aggregate magnetic B-Field (LABF) encompassing the SSO Earth-Sun local time of the descending note orbit evolution. Magnetic torque coils actuate the slew maneuver which is fully settled before the next ROI. The spacecraft is capable of slewing the inertial spin-axis orientation by 1.3 degrees in any direction during this maneuver.

### Eclipse

Each spacecraft sources power from its battery during eclipse by Earth. Minor oscillations of the booms and brackets due to the thermal change as the spacecraft enters or leaves eclipse are an order of magnitude below the allowable pointing control error.

### Downlink

Each spacecraft can downlink and receive pending MOC command uploads up to twice each day via the baseline Near Earth Network Direct-to-Earth services from ground stations located in Kiruna Sweden, Punta Arenas Chile, Santiago Chile, South Point Hawaii USA, North Pole Alaska USA, and Dongara Australia through the dual omni-directional S-band antennas. The spacecraft bus Level-0 comm-interface card initiates the CDPU to Forward transmit the instrument dataset, interleaved with spacecraft bus unique housekeeping VCDUs in the telemetry stream, relayed to the MOC in real time. All housekeeping telemetry has first priority in the downlink in the MOC for quick-look state of health verification. The remaining Science data follows and all MOC received Level-0 data is archived locally (for a temporary quality check period) and simultaneously mirrored to the master SOC archive. After verification of full transmit, or Loss of Signal (LOS), the downlink concludes and each TRACERS spacecraft is, once again, pre-positioned for the next ROI passage.

### Mission Ground Operations

TRACERS mission ground operations are concurrent with all operational modes once Science Main Mode has been activated. The SOC receives regular updates of newly acquired instrument housekeeping and science data. The housekeeping data and limit checks are distributed to each instrument node site for state of health concurrence. Any forecast long-term calibrations or operational updates, (MOC or MEB direct/derived telemetry limits updates, local aggregate magnetic B-Field target attitude vectors, MEB table parameters) are formed and relayed back to the SOC and MOC for the next update/uplink.

## Spacecraft

TRACERS consists of two nearly identical spacecraft, termed T1 & T2, that take advantage of Millennium Space Systems’ ALTAIR core bus platform. The spacecraft are passively spin-stabilized and operate at a nominal rate of 10 RPM. For TRACERS, the platform and overall vehicle design is optimized to provide substantial improvement in electrostatic and magnetic cleanliness to accommodate the instrumentation required for primary science objectives.

The primary structure of the TRACERS spacecraft employs an aluminum honeycomb composite face sheet panel construction connected to a series of machined aluminum frames. Most spacecraft components mount to the top and bottom decks, while select elements reside on side panels due to required fields of view and to optimize mass distribution for spin balance. The architecture allows for parallel integration and test of the overall vehicle avionics and propulsion system and provides flexibility in flow and access for testing.

TRACERS Telecommunications uses an S-band radio system for downlink and uplink with NASA Space Network (NSN) assets at a max rate of 6 Mbps and uplink rates of 32 kbps. The TRACERS S-band radio was upgraded for secure uplink, grounding, and increased data rates. Patch antenna placement provides near omni-directional coverage.

TRACERS incorporates a cold-biased thermal control system employing both passive and active techniques. The TRACERS Electrical Power System is composed of eleven solar array panels, lithium-ion batteries, and ALTAIR power management modules. Solar arrays are positioned on each of the 8 side panels and -Z decks for power generation. The spacecraft harnesses incorporate several unique features to ensure low magnetic properties and high electrostatic cleanliness for data & power lines.

TRACERS’ Hydrazine propulsion system is used for orbit maintenance, T1 to T2 separation adjustments, and de-orbit. TRACERS’ guidance and control mechanism adopts a spin-stabilized configuration, featuring periodic magnetic torque coil modulated attitude mini-slews, for local aggregate magnetic B-Field vector alignment and long-term nutation/spin trimming. The spacecraft relies on a Kalman-filter estimator, integrating a Sun sensor, ALTAIR standard inertial measurement units and three-axis magnetometers, and a GPS receiver to facilitate accurate attitude determination. This controller governs the operation of three magnetic torque coils for standard maneuvers to maintain nominal spin rate.

Millennium Space Systems’ heritage ALTAIR avionics manage command and data handling; thermal; guidance, navigation, and control; propulsion; and communication functions.

The Flight Software for TRACERS tailors the Millennium Space Systems’ ALTAIR heritage bus architecture platform to accommodate TRACERS’ specific requirements. This Flight Software undertakes critical tasks, such as device telemetry collection, fault detection and recovery, command formatting, routing, and downlink telemetry.

The TRACERS spacecraft integrate an onboard fault management system to ensure seamless nominal operations and proficiently manage fault detection and correction.

**Fig. 18:**
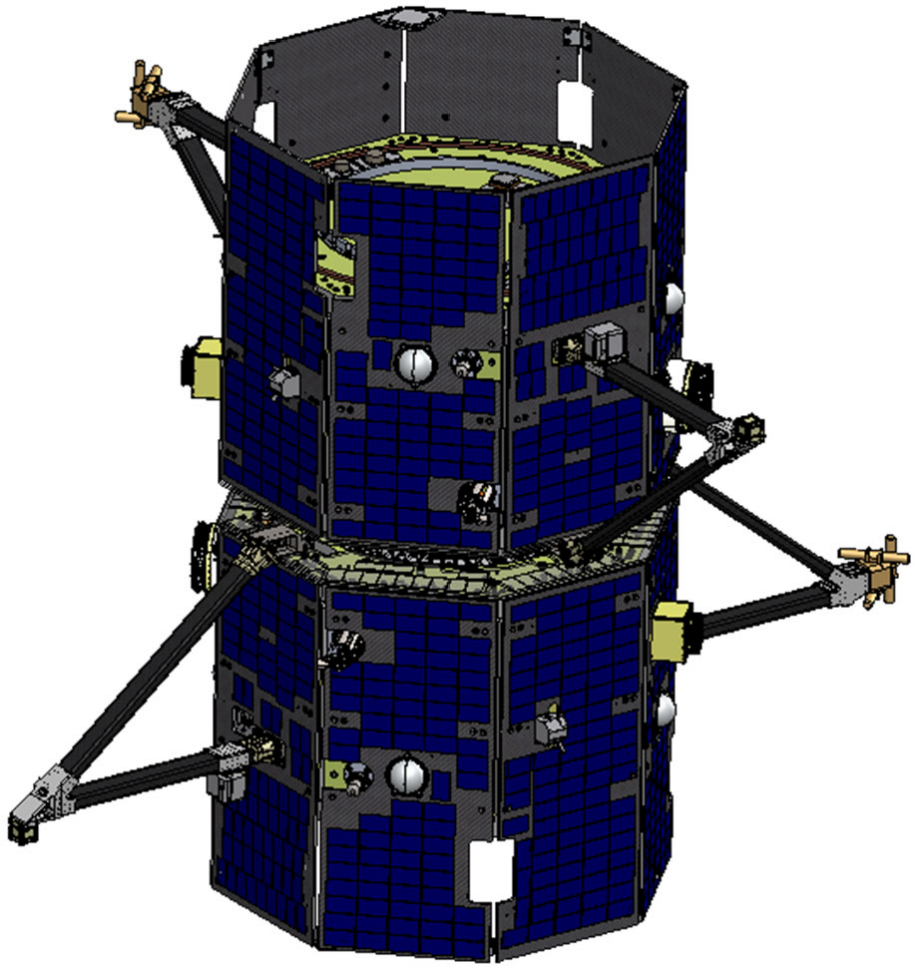
TRACERS launch configuration with the two-spacecraft stacked

The TRACERS vehicles will be launched in a stacked configuration as shown in Fig. 18 as a primary rideshare or “cake topper” on a SpaceX Falcon 9. Each TRACERS spacecraft has a not-to-exceed launch mass of 200 kg.

## Coordination with Ground Instruments

The northern and southern cusps are equally viable locations for TRACERS science. However, science data volume and spacecraft attitude reorientation requirements limit observations to one cusp per orbit. The northern cusp is used during the twelve-month primary mission because there are significant ground assets that contribute to this science enhancement option.

The 10:30 local time of the descending node of TRACERS’ Sun Synchronous Orbit (SSO) overflies Svalbard 1-2 times a day, providing an opportunity to connect with existing CUTLASS and EISCAT radar facilities and ground optical sites on Svalbard. Together, these ground-based facilities image auroral forms associated with cusp electrodynamics and electron precipitation to provide important measurement context. For the nominal launch date, there are over 500 predicted overflights of the island in twelve months.

The CUTLASS radar provides large scale imaging of the velocity structures over Svalbard. This coherent scatter radar has been used by TRACERS team members and others in the past for supporting rocket missions overflying Svalbard. The lower ionospheric diagnostics provided by CUTLASS helps confirm the location of the cusp, as well as its east-west boundaries to provide enhanced context for TRACERS observations.

The ground optical sites on Svalbard can image the poleward moving auroral forms (PMAF) and other auroral structures that are associated with cusp electrodynamics. The University of Oslo maintains a set of all-sky cameras that are ideally situated to observe the cusp region and electron precipitation features.

The EISCAT radar on Svalbard can provide detailed measurements of the ionospheric conditions that arise from poleward moving auroral forms precipitation. This radar can provide detailed density and temperature measurements of the ionosphere over Svalbard, enabling TRACERS to diagnose the observations of the cusp ion and electron precipitation more fully.

Conjugate measurements can also be provided by North American instruments such as radars (Greenwald et al. 1995), ground magnetometers (Mann et al. 2008), imagers (Donovan et al. 2006), and GNSS receivers (Jayachandran et al. 2009).

## Science Enhancement: TRACERS Student Rocket

The TRACERS Student Rocket mission, Observing Cusp High-altitude Reconnection and Electrodynamics (OCHRE), has the primary goal of training the next generation of space physicists and instrumentalists (Powers et al. 2025, this collection). The mission is largely led and managed by PhD students working from multiple institutions and managed by a postdoctoral research fellow with guidance from senior scientists. They will be responsible for many aspects of the final mission, including serving as instrument leads, Deputy PI for the student rocket, and supervising undergraduate students in instrument building and calibration.

The science goal of the student rocket is to characterize ion and electron distribution profiles and reconnection signatures in the cusp while utilizing contextual information from the TRACERS spacecraft mission flight to frame the observations. The launch will be coordinated to occur within ±45 minutes of a TRACERS’ cusp crossing during southward IMF conditions, to target an appropriate cusp event. The TRACERS Student Rocket will answer three primary science questions

1. To what extent do wave-particle interactions with broadband extremely low frequency (BBELF) waves dictate ion pitch angle distributions in the cusp?

2. Is there a prevailing spatial structure to ion outflows in the cusp?

3. Can high resolution measurements of the cusp electron edge describe the temporal variability in newly reconnected field lines?

OCHRE will be launched on a Black Brant XII from Andøya Space Center, Norway. With a payload consisting of a suite of instruments based on TRACERS heritage, the student rocket will be well equipped to answer the above science questions and supplement the broader TRACERS science goals. Details on the student rocket mission and science goals can be found in Powers et al. (2025, this collection)

Overall, the TRACERS student rocket is a ground-breaking student-led and student managed mission format that provides a ready training ground for undergraduate and graduate students in the design, fabrication, assembly, test, integration, launch, and data analysis techniques associated with flight hardware and in situ fields and particle measurements in the LEO environment. Working under the guidance of senior personnel, this mission aligns closely with NASA’s aspiration to make best use of the expertise and resources available in large missions to recruit and mentor future students, space scientists, aerospace engineers, and managers. It will address questions about the low-altitude nature of the cusp that TRACERS alone cannot determine, thus adding to the science enhancement of the main TRACERS mission and the goals of NASA’s Heliophysics Division.

## Operations and Data Availability

### Ground Data System

The ground data system comprises the TRACERS Mission Operations Center (MOC) and the TRACERS Science Operations Center (SOC). The MOC receives downlinked spacecraft telemetry (both instrument and spacecraft bus) from the Near Earth Network. The MOC passes these data to the SOC, which performs initial processing of science data from raw instrument telemetry to initial time-tagged (UTC) data; applies initial static and reversible calibrations to convert raw telemetry into pseudo-physical units; generates Quicklook plots; and then passes those data to the instrument teams for further processing.

### Mission Operations Center (MOC)

Millennium Space Systems (MSS) operates the TRACERS MOC. The MOC is the system of software, hardware and people that enables engineers to command the TRACERS spacecraft and analyze their telemetry. The MOC processes and tools were verified and validated during Mission Integration and Test. The MOC will then use these same procedures and tools during normal mission operations. These tools build spacecraft system commands, process and analyze telemetry, allow for telemetry to be queried and viewed, and allow for configuration files to be built and commands to be input. The tools also flow data to a variety of end users and clients, including science operations and science remote node clients, cognizant instrument, and spacecraft bus sustaining engineering staff, as well as data products for NASA (CARA, EHPD, the Near Earth Network, CP/NICS, SPDF and HQ) planning, validation, and mission support.

Instrument commanding is implemented by commands for each instrument being requested by the respective instrument teams, transmitted to the SOC which then packages and reviews them. and sends them to the MOC which validates them and then, if appropriate, commands the spacecraft. The MOC transmits commands and command sequences to the Near Earth Network for uplink to the spacecraft. Command and sequence generation is performed on MOC subsystem workstations for spacecraft level command and passed through the MOC flight operations file system for uplink. The TRACERS command and sequence generation and uplink process has operational flight heritage from the MSS Altair Pathfinder spacecraft.

All downlinked instrument data, both engineering telemetry and science data, comply with the CCSDS format for data packets and all spacecraft data comply with the CCSDS framing format. Spacecraft data are first received on the ground at the Near Earth Network and are then transmitted to the MOC. Each producer of data packets on the spacecraft has one or more Application Identifiers (APID’s) to identify the type of data it is creating. All engineering and science data are assigned an appropriate APID relative to their data type. Additionally, all data are stored onto the project server where they are available to the various mission teams for the duration of the project.

At the MOC, data packets containing spacecraft engineering telemetry are processed into key data that are displayed for real-time monitoring. Additionally, the MOC team monitors the data to perform telemetry trending analysis. All science and instrument engineering data are distributed to the SOC for science data processing at both the SOC and instrument team nodes.

### Science Operations Center (SOC)

The TRACERS SOC performs instrument science data processing in concert with the instrument teams. The SOC implements basic instrument-level processing to convert raw telemetry into properly time-tagged data, apply basic calibrations to convert to pseudo-physical units, and produce Quicklook plots. The SOC then passes the time-tagged and partially calibrated data to the instrument teams for further, higher level processing into fully calibrated data and derived data products via ISTP-compliant CDF files. Once processed by the instrument teams, these data files are transmitted back to the SOC which maintains the TRACERS data servers to provide data to the science team, science community, and general public.

The SOC is the centralized location, at the University of Iowa, for all TRACERS instrument data processing during the operational phases of the mission. Spacecraft telemetry, science instrument data, and spacecraft ephemerides from the MOC are received by the SOC, which sorts, validates, and packages telemetry data and inserts it into the SOC database.

TRACERS uses the NASA Spacecraft Planet Instrument C-matrix Events (SPICE) information system to handle orbit and attitude information in the science data processing. SPICE information is generated by the SOC and is available to data processing algorithms via the SOC data repository and to the instrument teams. Data are pulled from the database and data repository into the instrument-specific data processing pipelines at each instrument team’s home institution. The instrument-specific data processing pipelines return the calibrated data products to the SOC file server for dissemination to the general public and subsequent packaging and transfer to the SPDF.

The SOC is also responsible for generating, maintaining and archiving all SPICE information for the project. These data are held in the repository at the SOC and are made available to operations, instrument, and science teams as needed. The SOC is responsible for archiving the TRACERS SPICE repository along with all other observational data.

### Science Data Products

An Instrument Co-I leads each instrument team. It is the responsibility of these teams to develop, produce, and operate data processing pipelines for processing data and for delivering those data to the SOC. The instrument teams work with the SOC to coordinate these activities.

Each instrument team may change or update the calibration data or algorithms during the mission, leading to reprocessing and version-controlled release of updated data products. Additionally, the SOC maintains an archive of calibration data for use in final archiving. The validated data processing software is configuration-controlled and configuration-managed so that each data product has a known provenance.

The general philosophy for data product production follows the levels shown in Table 2. The SOC processes raw telemetry files from the MOC into L0 data which has had duplicated packets and packets with bad checksums removed. Those packets are then inserted into the packet telemetry database.

**Table 2:** TRACERS data product levels, responsible parties, latency, and distribution

	Level	Description	Code Execution	Target Nominal Latency	Latency Requirement	Distribution
INTERNAL/NOT FOR PUBLICATION	0	Received CCSDS with duplicate/corrupt packets removed.	SOC	< 1-hour	24-hours	SOC
		On-demand conversion of L0 packets for rapid visualization.	SOC	On-demand	On-Demand	TRACERS Internal
		Coarse physical units possible if calibrations loaded into the tool.				
	1a	Unpacked and ordered data from CCSDS packets.	SOC	< 3-hours	48-hours	TRACERS Internal
		All data in as-received engineering units in sensor frame.				
		Best known timing (bus time + known offsets) on each sample.				
		All relevant header metadata propagated.				
	1b	Coarse physical units based on static and reversible calibrations.	SOC	< 24-hours	7-days	TRACERS Internal
		Known lost/corrupted data filled/flagged as appropriate.				
		Spacecraft frame and common preliminary de-spun coordinates based on preliminary as-flown SPICE Kernel.				
		No convolution of data from multiple instruments.				
		Definitive SPICE Kernel				
	QL	Automatic .png plots generated on-receipt of L1b data.	SOC	< 24-hours	7-days	TRACERS Internal
		Plot-per-orbit and plot-per-day to assess instrument health.				
		Plot-per-orbit and plot-per-day to identify potential science.				
PUBLIC	2	Calibrated physical units (via in-situ calibration if applicable).	Instrument Team	< 2-weeks	1-month	Public
		Spacecraft and geophysical frame (including field aligned) using the current definitive as-flown SPICE Kernel.				
	3	Derived by irreversibly combining data from multiple instruments.	Instrument Team	< 1-month	2-months	Public
		Derived by irreversibly transforming/reducing data using physics.				
	4	Derived by irreversibly integrating data from models	Modelling Team			

The Science Operations Center then creates L1a data by unpacking and ordering the CCSDS packets from L0 and applying the best-known time to each sample. All data at L1a are in the as-received engineering units in their native instrument coordinate frame. All relevant header metadata are propagated from L0 to L1a.

The SOC then creates L1b data by applying static and reversible calibrations, defined by the instrument/subsystem teams, to convert measurements into pseudo-physical units. These calibrations are version-controlled within the SOC and are traceable to subsequent products. Known data gaps are reported in the output products. Data are converted to the common spacecraft frame TRACERS Spacecraft Coordinate System (TSCS) via static SPICE transforms, and into a common preliminary de-spun coordinate frame based on the as-flown SPICE kernel. Data from multiple instruments will not be convolved to create L1b products. L1b data are saved into CDF files which serve as the hand-off point from the SOC to the cognizant instrument/subsystem teams for further processing.

The SOC automatically generates QuickLook plots from the L1b data to facilitate the rapid evaluation of instrument health and initial visual detection of scientifically promising measurements.

L2 data are scientifically usable, calibrated data products. For example, magnetometer or E-field data in the TSCS or particle counts in energy (or energy step) and pitch angle. L2 and higher-level data products may also be delivered in a common de-spun coordinate and/or geophysically relevant coordinate frames. The generation of L2 products may require spacecraft ephemeris and attitude data, and/or data from other instruments. The L3 data are derived by irreversibly combining data from multiple instruments and/or transforming/reducing data using physics (for example, estimating field aligned current from the perturbations of the measured magnetic field). Level 4 data are derived by irreversibly integrating data from models.

The instrument team pipelines poll the SOC server for new data files, and then pull instrument data and necessary ancillary data from the file system to the instrument home institutions to begin processing. Each data product pipeline flags suspect or missing data intervals to notify the higher-level software tools.

### Data Availability

The high-level data products that are intended for use by the scientific community are shown in Table 3.

**Table 3:** TRACERS High Level Data Products

Instrument(s)	Level: Data	Description
ACI	L2: Ion Energy Instantaneous Pitch/Look Angle Distributions	Differential Energy Flux {Energy, Pitch Angle/Look Direction, Time}
	L3: Ion Energy-Pitch Angle Averaged Distributions	Differential Energy Flux {Energy, Pitch Angle/Look Direction, Time}
ACE	L2: Electron Energy Instantaneous Pitch/Look Angle Distributions	Differential Energy Flux {Energy, Pitch Angle/Look Direction, Time}
	L3: Electron Energy-Pitch Angle Averaged Distributions	Differential Energy Flux {Energy, Pitch Angle/Look Direction, Time}
MAG	L2: DC Vector Magnetic Field	Geophysical Magnetic Field in geophysical coordinate system
	L3: Vector Magnetic Perturbations	Geophysical Magnetic Field minus model in geophysical coordinates
	L3: Field Aligned Currents	Derived from Vector Magnetic Perturbations
EFI	L2: Perpendicular DC Electric Field	Electric Field in geophysical coordinate system, with *V* × *B* removed
	L2: Wave AC Electric Field	AC Electric Field in geophysical coordinate system, with *V* × *B* removed
	L3: Plasma Density from Wave Measurements	HF receiver reduced to density
MSC	L2: Wave AC Magnetic Field	AC Magnetic Field in geophysical coordinate system
MAG/MSC	L3: Wave AC Magnetic Field	Combined MAG and MSC
MAG/MSC/EFI	L3: Wave Poynting Flux	Combined MAG, MSC, EFI

The TRACERS schedule for data products is based on mission start combined with when telemetry is received on the ground. For the first 3 months of TRACERS mission operations, no data are publicly released. This allows time for the TRACERS team to properly verify on-orbit instrument operations, data processing pipelines, and calibrations to ensure that quality data are released to the scientific community and general public. After this initial period of data and processing verification, the SOC begins making available scientifically usable TRACERS L2 data from the start of the mission, transitioning over a 3-month period to producing L2 data products that are released to the SPDF 1 month after receipt by the SOC. L3 products will be released to the SPDF 2 months after receipt by the SOC, and L4 products will target release for 3 months after receipt by the SOC; this may extend depending on the complexity of the data product. L1a/b data products are delivered to the SPDF for archiving at the end of mission phase.

## Summary

The overarching science goal of the TRACERS mission is to connect the cusp to the magnetosphere by discovering how spatial or temporal variations in magnetic reconnection drive cusp dynamics. This goal will be achieved using two identical, small satellites in a common low-Earth orbit in a follow-the-leader configuration. TRACERS will make repeated plasma and fields measurements in the cusp that are analyzed using established dual-satellite techniques and supported by modeling that ensures science closure. The TRACERS hardware is provided by collaborations between the University of Iowa, Southwest Research Institute, University of California Los Angeles, University of California Berkeley and Millennium Space Systems. The larger science team consists of experts in reconnection, cusp physics, and modeling. TRACERS will complete its primary science objectives during a twelve-month baseline mission. The TRACERS instrument suite and spacecraft provide a robust, highly capable plasma science package that can be leveraged for a wide range of future space physics investigations.
